# Distinct neuronal vulnerability and metabolic dysfunctions are characteristic features of fast-progressing Alzheimer's patients with Lewy bodies

**DOI:** 10.1016/j.jbc.2025.108396

**Published:** 2025-03-10

**Authors:** Mohammed Waseequr Rahman, Preeti Sharma, Trisha Chattopadhyay, Sivaprakasam R. Saroja

**Affiliations:** 1 Center for Brain Research, Indian Institute of Science, Bangalore, India; 2 Manipal Academy of Higher Education, Manipal, Karnataka, India

**Keywords:** Alzheimer's diseas, tau pathology, Lewy bodies, dementia progression, metabolic dysfunctions

## Abstract

Tau protein accumulation is linked to dementia progression in Alzheimer's disease (AD), with potential co-pathologies contributing to it. The progression of dementia in patients with AD varies between individuals, and the association between co-pathology and heterogeneity in dementia progression rate remains unclear. We used longitudinal cohort data, *postmortem* brain tissues, and biochemical methods such as immunoassays and proteomic profiling to investigate the molecular components associated with progression rate. We report that AD with comorbidities, such as dementia with Lewy bodies (DLB) and TDP-43 pathology, progress faster than AD alone. Patients with AD-DLB had higher levels of soluble oligomeric tau proteins and lower levels of insoluble tau proteins compared to those with AD alone. Our data suggest that α-synuclein fibrils may enhance tau aggregation through cross-seeding. The prefrontal cortex is more vulnerable to Lewy body pathology than the temporal cortex, and Tau and α-synuclein aggregate in distinct neuronal populations, indicating selective neuronal and regional vulnerability to their respective pathologies. Dysfunctional metabolic pathways were more strongly associated with patients having fast-progressing AD-DLB. Our study suggests that comorbidities, such as α-synuclein aggregation and metabolic dysfunctions, are associated with rapidly progressing AD patients, highlighting the importance of patient subgrouping for clinical trials.

Alzheimer's disease (AD) is the leading cause of dementia, characterized by progressive neurodegeneration along with the aggregation of amyloid and tau proteins (1, 2, 3). Tau pathology, in the form of neurofibrillary tangles (NFTs), strongly correlates with cognitive decline (4). Tau accumulation begins in the entorhinal cortex, followed by limbic regions, and subsequently spreads across the neocortical areas, leading to progressive cognitive decline (3, 5). Tau protein is predominantly expressed in neurons and plays a key role in microtubule stability (6, 7, 8, 9). In adult human brains, tau exists in six isoforms, classified as 3R and 4R isoforms based on the number of microtubule-binding repeats (10). In AD, tau is hyperphosphorylated (11), causing it to detach from the microtubule and form oligomers (12, 13, 14).

The rate of cognitive decline varies among AD patients (15). Some AD patients experience rapid cognitive deterioration, while others experience a slower decline, living only with mild cognitive impairments for decades (16). It is hypothesized that this heterogeneity in dementia progression may be due to several underlying co-pathologies (15, 17). Various proteinopathies, such as α-synucleinopathies, TAR DNA binding protein-43 (TDP43) proteinopathies, and others, overlap with typical AD pathologies (17, 18). α-synuclein pathology is present in nearly 50% of AD cases upon autopsy (19). Among these, dementia with Lewy bodies (DLB) is one of the major α-synucleinopathies and is the second leading cause of proteinopathic neurodegenerative dementia after AD (20). α-synuclein, a presynaptic protein of unclear function, forms aggregate in neurons of the neocortex, midbrain, and brainstem, manifesting as Lewy bodies and Lewy neurites in DLB (21). The presence of phosphorylated α-synuclein in the aggregates of the DLB brain is evident, making it a key marker for Lewy body pathology in DLB (22, 23).

To determine whether any specific co-pathology/co-pathologies accelerate dementia progression in patients with AD, we first utilized clinical data obtained from an AD longitudinal cohort study. Our data analysis showed that patients with AD having co-pathologies such as AD-DLB and AD-TDP43 progress rapidly on the Mini-Mental State Examination (MMSE) dementia rating scale compared to AD-alone and AD with all other co-pathology subgroups. Given that the number of patients with AD-DLB is greater than that of patients with AD-TDP43 in the cohort, we used the post-mortem brains of patients with AD-DLB for subsequent analysis to understand their biochemical and molecular heterogeneities, comparing AD-alone and normal controls. We found that fast-progressing AD-DLB had lower levels of sarkosyl-insoluble tau. In contrast, total soluble oligomeric tau/high molecular weight (HMW) tau, as well as the number of cells bearing aggregate burden, were higher in fast-progressing AD-DLB than in slow-progressing AD-alone patients. AD-DLB patients also exhibit selective neuronal and regional vulnerabilities to NFT and Lewy body aggregation. Furthermore, patients with fast-progressing AD-DLB show more severe metabolic dysfunctions compared to patients with slow-progressing AD alone.

## Results

### The dementia of Alzheimer's patients with DLB and TDP-43 progresses faster

**Figure 1:**
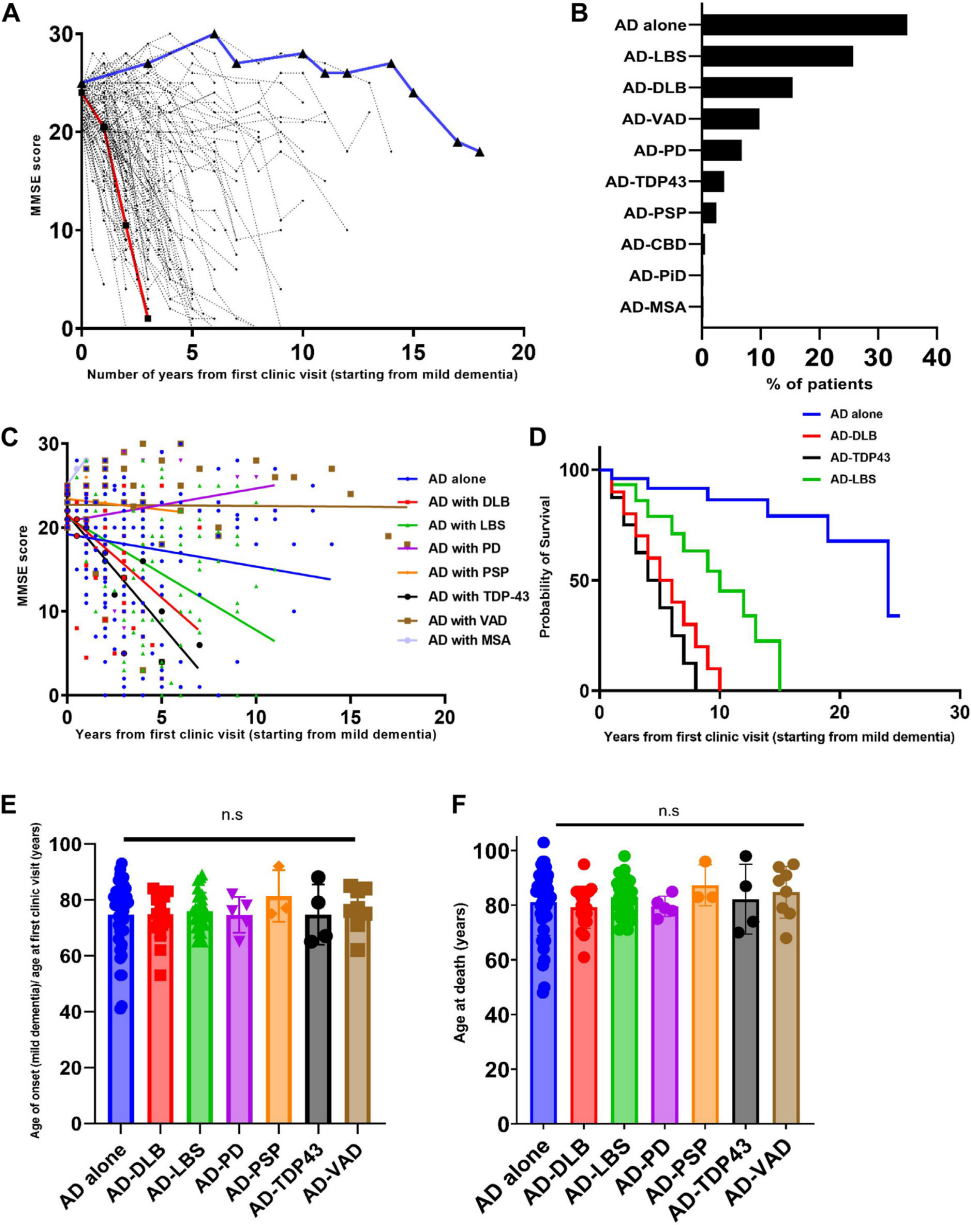
**Analysis of longitudinal data from the cohort.***A*, spaghetti plot representing all the patients with AD from the cohort who were tracked longitudinally on the MMSE scale since their first clinic visit (with mild dementia, MMSE 25–20) over the years of disease duration until their death or clinical withdrawal. The *red* and *blue* connecting lines highlight the individual patients of fast and slow dementia progression, respectively. (n = 120). *B*, types of co-pathologies present among patients with AD and their percentage of occurrence in the longitudinal cohort without any segregation. (Total number of patients: AD-alone = 129, AD-DLB = 57, AD-LBS = 95, AD-VAD = 36, AD-PD = 25, AD-TDP43 = 14, AD-PSP = 9, AD-CBD = 2, AD-PiD = 1, and AD-MSA = 1). *C*, the Spaghetti plot represents the longitudinal cohort where patients with AD were segregated based on mild dementia (MMSE 25–20) onset at their first clinic visit along with their co-pathological presentation. All the groups were linearly fit using simple linear regression. *D*, Kaplan-Meier survival analysis of dementia for AD-alone, AD-DLB, AD-TDP43, and AD-LBS (all four groups have their slopes significantly deviated from zero in Fig. 1*C*). Comparison of survival curves- Log-rank (Mantel-cox test), *p* value= <0.0001; Log-rank test for trend, *p* value= <0.0006; Gehan-Breslow-Wilcoxon test, *p* value= <0.0001. *E*, the age of all patients at their first clinical visit presenting with mild dementia (MMSE: 20–25) was included in this analysis. *F*, the age at death of all patients presenting with mild dementia (MMSE: 20–25) at their first clinical visit was included in this analysis. For both ‘E’ and ‘F’, Kruskal-Wallis one-way ANOVA was performed, followed by Dunn's multiple comparisons, which indicated no significant differences among the groups. For ‘*E*’- Kruskal Wallis ANOVA test (*p* = 0.9583); ‘*F*’- Kruskal Wallis ANOVA test (*p* = 0.6177). Sample sizes for panels ‘*A*,’ ‘*C*,’ and ‘*D*’ are as follows: AD-alone = 43, AD-DLB = 18, AD-LBS = 35, AD-VAD = 8, AD-PD = 5, AD-TDP43 = 4, AD-PSP = 3, AD-CBD = 2, AD-PiD = 1, AD-MSA = 1. For panels ‘*E*’ and ‘*F*,’ sample sizes are as follows: AD-alone = 43, AD-DLB = 18, AD-LBS = 35, AD-VAD = 8, AD-PD = 5, AD-TDP43 = 4, AD-PSP = 3. n.s. = non-significant.

We examined longitudinal data from patients with AD tracked over time using the Mini-Mental State Examination (MMSE) scale from the Arizona Study of Aging and Neurodegenerative Disorders/Brain and Body Donation Program. The spaghetti plot illustrates the heterogeneity in dementia progression of patients with AD who progressed from mild dementia (MMSE: 21-25) to severe dementia (MMSE: <12) at different rates (Fig. 1*A*). To illustrate this dementia progression variability, we randomly selected two patients: the red line shows an AD patient worsening to severe dementia in 5 years, while the blue line represents a patient with AD who never reached the severe dementia stage (Fig. 1*A*). Given that Alzheimer's disease frequently coexists with other pathologies (24), we next investigated the relationship between co-pathology and heterogeneity in dementia progression. Our analysis of the entire longitudinal dataset revealed varying frequencies of co-pathologies, including DLB (15.45%), TDP-43 (3.79%), Parkinson's disease (PD) (6.78%), Lewy body spectrum (LBS) (25.75%), progressive supranuclear palsy (PSP) (2.44%), vascular dementia (VaD) (9.76%), multiple system atrophy (MSA) (0.27%), and AD-only/alone (34.96%) (Fig. 1*B*). We subsequently selected all Alzheimer's disease patients with mild dementia (MMSE scores of 20–25) on their first clinical visit from the longitudinal study for our further analysis. Patients were then subcategorized based on their co-pathological presentations from available post-mortem data. Co-pathology-based linear and Kaplan-Meier survival analyses further demonstrated that AD-DLB and AD-TDP-43 progressed more quickly than other groups, including AD-only/alone (Fig. 1, *C* and *D*). The faster dementia progression could be due to their prolonged dementia period or delay in the onset of dementia itself. To address this, we analyzed the age at the onset of mild dementia or the age at the first clinical visit presenting mild dementia. The analysis revealed no significant differences across the stratified groups, with all the groups showing a similar age at the onset of mild dementia (Fig. 1*E*). We then assessed the age at death across all groups and found no significant differences, as all groups had a similar age at the time of death (Fig. 1*F*). These findings indicate that the duration of dementia from onset to death is comparable across all groups. However, despite this similarity in dementia duration, patients with AD having concomitant DLB and TDP-43 co-pathologies tend to experience faster progression of dementia. Together, our analysis demonstrates that DLB and TDP-43 co-pathologies are associated with the rapid progression of dementia in patients with AD.

### Differential tau burden in fast (AD-DLB) and slow (AD-only/alone) dementia progressors

**Figure 2:**
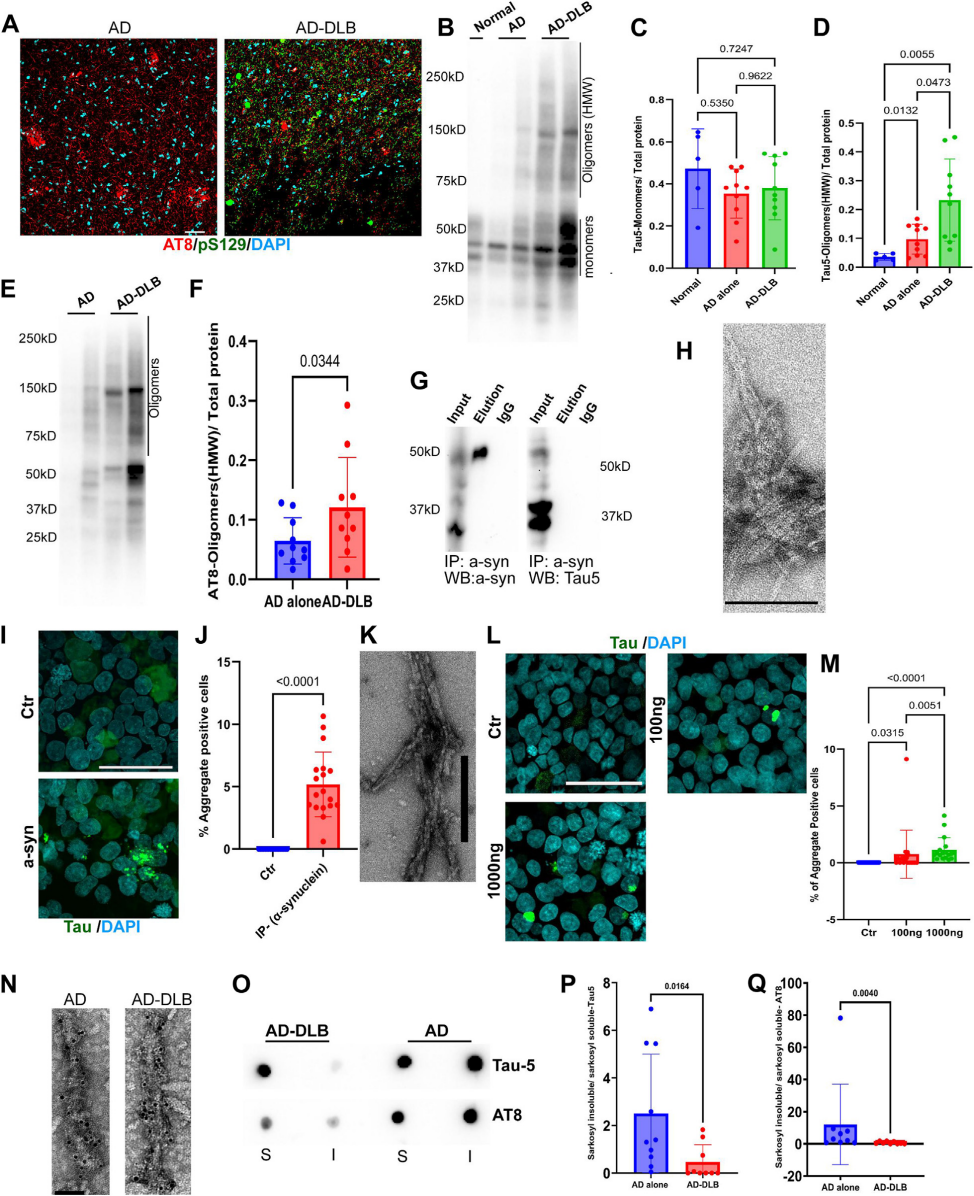
**Examination of tau species from slow-progressing (AD alone) and fast-progressing (AD-DLB *postmortem* brain tissues**. *A*, representative confocal images of frozen human brain *postmortem* tissues (BA20) from AD alone and AD-DLB, processed for IHC and stained with AT8 (p-Tau) antibody marker for tau pathology, the p-α-synuclein (pS129) antibody marker for DLB pathology, and DAPI, a nuclear stain (scale bar = 50 μm). *B*, a representative Western blot image from a PBS soluble fraction (BA20) was probed with Tau5 antibodies. *C*, group differences for Tau5-Monomers, normalized by total protein, were assessed using the Brown-Forsythe ANOVA (*p* = 0.4189) and Welch's ANOVA (*p* = 0.4877), followed by Dunnett's T3 multiple comparisons test. Here, immunoreactive bands ranging from 50kD to 37kD were considered as monomers. Normal = 5, AD alone = 10, AD-DLB = 10. *D*, group differences for Tau5-Oligomers (HMW) tau, normalized by total protein, were assessed using the Brown-Forsythe ANOVA (*p* = 0.0019) and Welch's ANOVA (*p* = 0.0005) followed by Dunnett's T3 multiple comparisons test. Here, an immunoreactive band of above 50 kD was included in the quantification, representing oligomeric (HMW) tau. Normal = 5, AD alone = 10, AD-DLB = 10. *E*, representative Western blot image from the PBS-soluble fraction probed with AT8 (p-Tau) antibodies. *F*, unpaired *t* test for AT8 (AT8-Oligomers) normalized by total protein. AD alone = 10, AD-DLB = 10. *G*, Western blot analysis of the immunoprecipitation (IP) fraction obtained using the p-α-synuclein (pS129) antibody from AD-DLB brain tissue (BA20). The IP fraction was probed with total α-synuclein (for IP) and tau5 (for co-IP) antibodies. The input lane represents the total protein fraction, the elution lane represents the α-synuclein-enriched IP fraction, and the IgG lane represents the eluted antibody control. *H*, representative transmission electron microscopy (TEM) image of α-synuclein fibrils isolated from AD-DLB brain. Scale bar = 100 nm. *I*, representative confocal images of the α-synuclein cross-seeding assay in Tau FRET biosensor cells using immunoprecipitated α-synuclein. Scale bar = 50 μm. *J*, quantification of the percentage of aggregate-positive cells in the α-synuclein cross-seeding assay shown in (*I*). Statistical analysis was performed using a Mann whitney's U test (*p* < 0.0001). Each data point represents the percentage of aggregate-positive cells from a single confocal image, with data collected from three independent experimental replicates. *K*, representative TEM image of commercially available α-synuclein preformed fibrils (PFFs). Scale bar = 200 nm. *L*, representative confocal images of the α-synuclein cross-seeding assay in Tau FRET biosensor cells using α-synuclein PFFs. Scale bar = 50 μm. *M*, quantification of the percentage of aggregate-positive cells in the α-synuclein cross-seeding assay shown in (*L*). Statistical analysis was performed using the Kruskal-Wallis test (*p* < 0.0001), followed by Dunn's multiple comparison tests. Each data point represents the percentage of aggregate-positive cells from a single confocal image, with data collected from three independent experimental replicates. N, Representative Tau5-immunogold TEM images of sarkosyl insoluble tau from AD and AD-DLB *postmortem* brains (BA20). (scale bar = 50 nm). *O*, Representative dot blot of sarkosyl-soluble (S) and sarkosyl-insoluble (I) fractions probed with Tau-5 and AT8 from AD-alone and AD-DLB *postmortem* brain (BA20). *P*, unpaired *t* test of sarkosyl-insoluble Tau-5 normalized by sarkosyl-soluble Tau-5 of AD alone (n = 10) and AD-DLB (n = 9). *Q*, unpaired *t* test of sarkosyl-insoluble AT8 was normalized by sarkosyl-soluble AT8 of AD alone (n = 9) and AD-DLB (n = 9).

Given that the frequency of AD-DLB was four times higher than AD-TDP43 (Fig. 1*B*) and there was no significant difference in dementia progression between both groups (Fig. 1*D*), we selected AD-DLB for further analysis. The details of the post-mortem brain tissues used are provided in the (Table S1). Using post-mortem brain tissues from the inferior temporal gyrus (Brodmann area 20/BA20), we validated the presence of AT8 (p-Tau) in AD-DLB and AD-only/alone and pS129 (p-α-synuclein) in AD-DLB (Fig. 2*A*). We next assessed the tau burden between the normal, slow-progressing (AD-only/alone) and fast-progressing dementia (AD-DLB) groups. We analyzed the total tau of PBS-soluble fractions from tissue homogenates using Western blot with the Tau5 antibody (Figs. 2*B* and S1*A*). The analysis revealed elevated levels of PBS-soluble oligomeric/high molecular weight (HMW) tau (>50 kD) in the AD-DLB group compared to AD alone and normal controls (Fig. 2*D*). However, no significant differences were observed in monomeric (50–37kD) and fragmented (25kD and below) tau levels across the groups (Figs. 2*C* and and S1*B*). Additionally, comparing oligomeric/HMW phosphorylated tau (p-tau; AT8) levels among normal, AD alone, and AD-DLB groups showed significant differences between normal and AD alone, as well as normal and AD-DLB, in multiple comparison tests. However, no significant differences were observed between AD alone and AD-DLB in the multiple comparison test, following Welch's ANOVA (Fig. S1, *C* and *D*). Interestingly, when we compared only two groups (AD alone and AD-DLB) with an unpaired *t* test, it revealed higher levels of oligomeric/HMW phosphorylated tau (p-tau; AT8) in AD-DLB compared to AD alone (Figs. 2, *E* and *F*, and S1*C*). Together, these findings indicate that while monomeric tau protein levels were similar across groups, oligomeric/HMW-total tau, and oligomeric/HMW-p-tau levels were elevated in AD-DLB compared to AD alone.

Unlike AD alone, AD-DLB exhibits an additional aggregate burden of α-synuclein pathology along with tau and Aβ. To investigate whether α-synuclein fibrils promote tau aggregation through cross-seeding, we first immunoprecipitated (IP) α-synuclein from AD-DLB *postmortem* brain tissue (BA20) and validated the immunoprecipitated fraction using total α-synuclein (for IP) and tau5 (for Co-IP) antibodies on a Western blot (Fig. 2*G*). The data showed the presence of α-synuclein, while tau proteins were absent in the elution. We further imaged the elution with TEM and confirmed the presence of α-synuclein fibrils (Fig. 2*H*). This IP fraction of α-synuclein fibrils from the AD-DLB brain was subjected to brief sonication, as described in the materials and methods, and tested for seeding activity in Tau FRET biosensor cells. The tau seeding experiment demonstrated the ability of α-synuclein fibrils from the AD-DLB brain to cross-seed endogenous tau fusion proteins (Fig. 2, *I* and *J*). To further validate the cross-seeding potential of α-synuclein fibrils, we used commercially available α-synuclein preformed fibrils (PFFs), which were validated by TEM (Fig. 2*K*). The α-synuclein PFFs were subjected to brief sonication, as described in the materials and methods, and tested for seeding activity in Tau FRET biosensor cells. We observed a concentration-dependent increase in endogenous tau cross-seeding (Fig. 2, *L* and *M*). Furthermore, we identified a positive correlation between p-tau and p-α-synuclein pathology levels in AD-DLB *postmortem* tissue (Fig. S2, *A* and *B*). However, α-synuclein, prepared according to the previous report, did not induce tau aggregation as described (25) (Fig. S3*A*). Together, these results highlight the potential synergy between tau and α-synuclein pathologies and the ability of α-synuclein fibrils to cross-seed endogenous tau. This interaction may contribute to the accelerated dementia progression observed in AD-DLB compared to AD alone, which lacks α-synuclein co-pathology.

Next, we analyzed insoluble tau from AD alone and AD-DLB groups, as insoluble tau aggregates are closely linked to neurofibrillary tangle formation. To examine the levels of insoluble tau, we isolated the sarkosyl insoluble fraction from the *postmortem* tissues of both groups using the standardized protocol (26). The presence of fibrils in the insoluble fractions was validated by immunogold transmission electron microscopy using the Tau5 antibody (Fig. 2*N*). We performed the dot blot analysis on the isolated sarkosyl insoluble fractions and found reduced total (Tau5) and p-Tau (AT8) in AD-DLB compared to AD-only/alone patients (Figs. 2, *O*–*Q* and S3*B*). These results suggest that AD-DLB exhibits reduced levels of total and phosphorylated tau in the insoluble fraction compared to AD alone. We then explored how tau and α-synuclein aggregates differ in their resistance to proteolytic degradation to understand the structural stability of these sarkosyl-insoluble fractions in fast dementia progressors. We performed a Pronase resistance assay using the AD-DLB insoluble fraction and found that tau aggregates exhibited greater resistance to Pronase digestion than α-synuclein aggregates. (Fig. S4*A*). Following our analysis of tau and α-synuclein aggregation, we next evaluated the differences in Aβ42 levels using an Aβ42-specific ELISA. Our analysis revealed no significant differences in Aβ42 levels in the GnHCl-soluble fraction isolated from post-mortem brain tissues (BA20 region) of individuals with AD-DLB compared to those with AD-only/alone (Fig. S4*B*). Together, our data show that patients with AD-DLB have more soluble tau oligomers/HMW tau, lower insoluble tau fibrils and an extra intracellular aggregate burden of α-synuclein with the ability to cross-seed endogenous tau.

### Distinct neuronal and regional vulnerability for tau and **α** synuclein aggregation in AD-DLB

**Figure 3:**
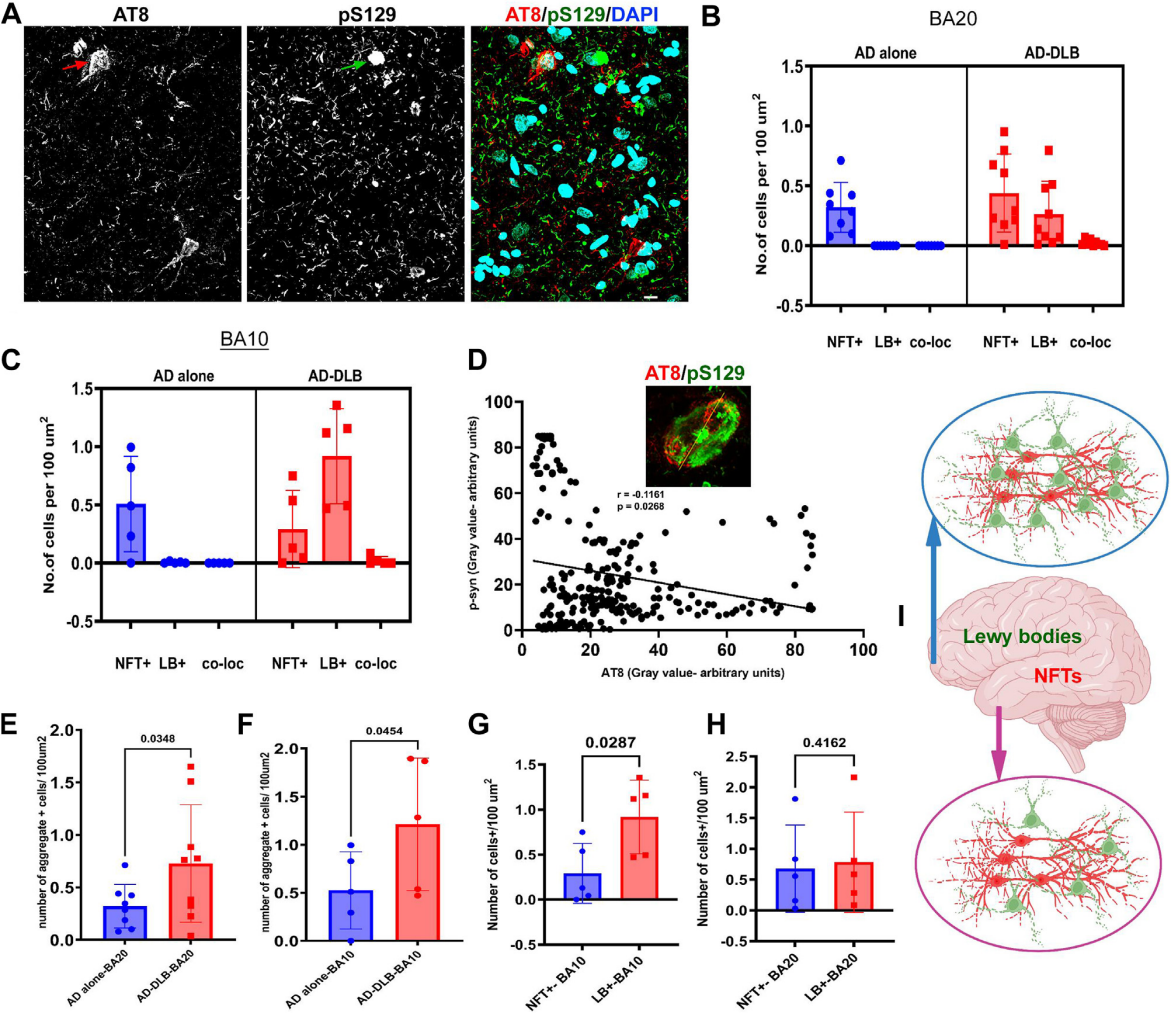
**Neuronal and regional vulnerabilities for NFTs and Lewy bodies.***A*, representative image of IHC (BA20) indicating different cell populations aggregating NFTs (AT8) and Lewy bodies (pS129). DAPI, a nuclear stain. scale bar = 10 μm. *Arrowheads* represent differential cellular vulnerabilities for NFTs and Lewy bodies *B*, quantification of NFT+, LB+, and co-localized cells (NFT+ & LB+) from AD alone (n = 8) and AD-DLB (n = 9) from the temporal cortex (BA20). *C*, quantification of NFT+, LB+, and co-localized cells (NFT+ & LB+) from AD alone (n = 5) and AD-DLB (n = 5) from the prefrontal cortex (BA10). *D*, co-localization analysis using Fiji-ImageJ intensity plots for AT8 (*red*) and p-α-synuclein (*green*) channels, scale bar = 7 μm. Spearman correlation between gray values of p-Tau (arbitrary units) and p-α-synuclein (arbitrary units), r = −0.1161, *p* = 0.0268. *E*, unpaired *t* test for total intracellular aggregate + cells (NFTs+ Lewy body cells) between AD alone (n = 8) and AD-DLB (n = 9) in the temporal cortex (BA20). *F*, unpaired *t* test for total intracellular aggregate + cells (NFTs + Lewy body cells) between AD alone (n = 5) and AD-DLB (n = 5) in the prefrontal cortex (BA10). *G*, unpaired *t* test between NFT+ and Lewy body + cells of the prefrontal cortex (BA10) from AD-DLB (n = 5). *H*, unpaired *t* test between NFT+ and Lewy body + cells of the temporal cortex (BA20) from AD-DLB (n = 5). *I*, representative diagram of NFT+ and Lewy body + cell burden in the temporal (BA20) and prefrontal cortices (BA10) of brain of patient with AD-DLB. This depicts differential neuronal and regional vulnerabilities for NFTs and Lewy bodies aggregation within AD-DLB disease. The image was created using Biorender.

Neurofibrillary tangles are known to aggregate in excitatory neurons in the AD brain (27, 28). Given that AD-DLB is characterized by both NFTs and Lewy body aggregates, we investigated whether NFTs and Lewy bodies co-aggregate and co-localize within the same neurons in the neocortex of AD-DLB patients. Interestingly, our findings revealed that NFTs and Lewy bodies aggregate in different cellular populations with low co-localization (<7% of cells) both in temporal (inferior temporal gyrus (BA20)) and prefrontal (anterior prefrontal cortex (BA10)) cortical regions (Fig. 3, *A*–*C*). The data further showed that even in those few co-localized cells, the spatial overlap between the aggregates was low (Fig. 3*D*). A high-magnification 3D view revealed spatially distinct tau and synuclein aggregates (Supporting Video). Our study revealed that AD-DLB patients contained considerably more total aggregate + cells (NFT positive + Lewy body positive cells) than those with AD alone, in both the temporal and prefrontal cortices (Fig. 3, *E* and *F*). However, the prefrontal cortex, rather than the temporal cortex, is more vulnerable to Lewy body + cells in AD-DLB (Fig. 3, *G* and *H*). Our study thereby indicates the presence of selective neuronal and regional vulnerabilities to tau and α-synuclein aggregation (Fig. 3*I*).

### Unique proteomic signatures of patients with fast and slow dementia progressing AD

**Figure 4:**
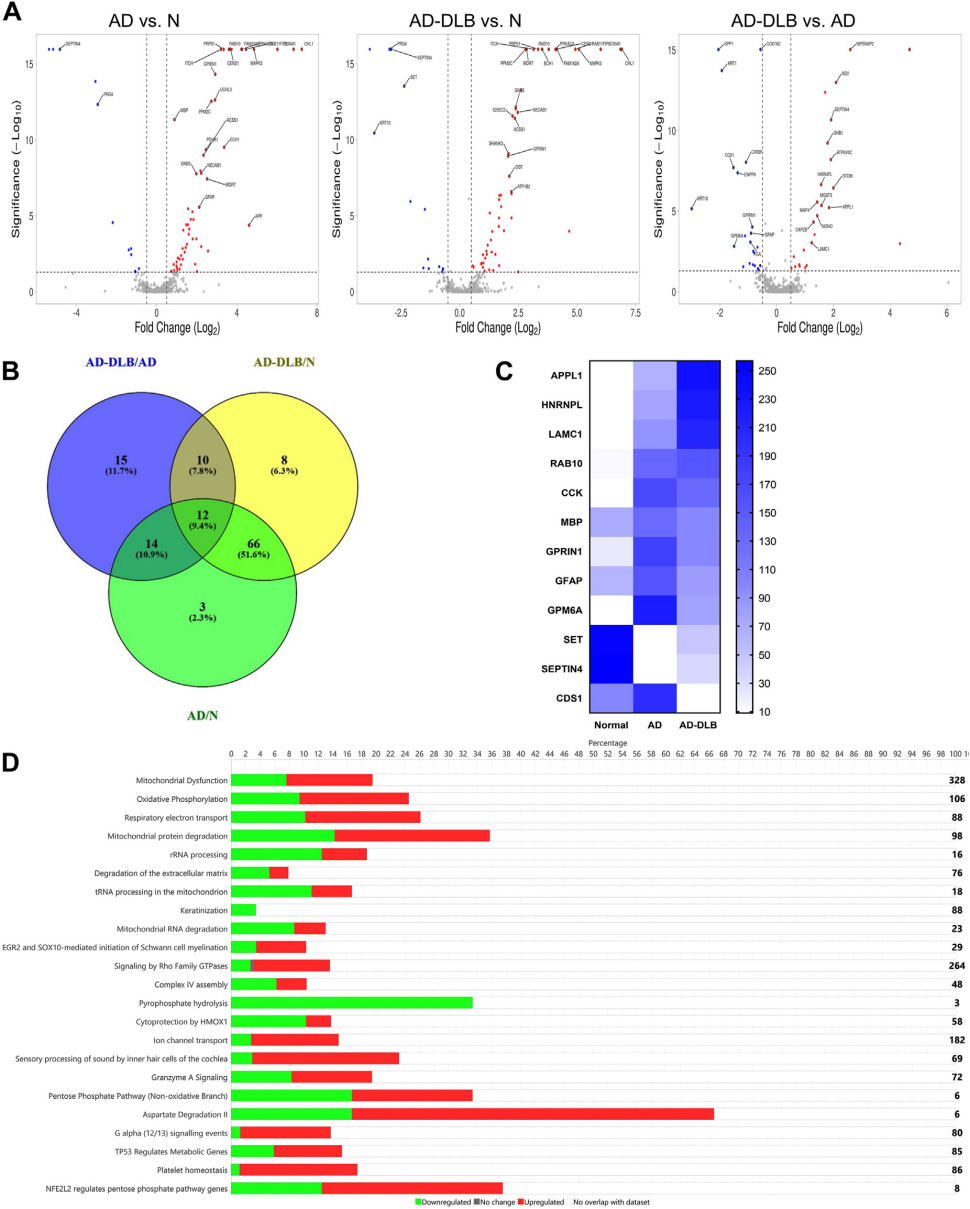
**Unique proteomic signatures and IPA pathway analysis of fast- and slow-progressing AD patients from their temporal cortex (BA20).***A*, Volcano plots showing significantly upregulated and downregulated proteins between pairwise comparisons of groups [thresholds set at log_2_FC ≥ ±-1.0 for biologically significant changes and -log_10_(q) ≥ 1.3 (q < 0.05) for statistical significance]. The volcano plots were generated by the VolcaNoseR package (https://huygens.science.uva.nl/VolcaNoseR/). Significantly upregulated proteins are highlighted in *red*, and significantly downregulated proteins are highlighted in *blue*. *B*, a Venn diagram showing the overlap of significantly dysregulated proteins between all pairwise comparisons of groups. The Venn diagram was created using Venny 2 (https://bioinfogp.cnb.csic.es/tools/venny/index2.0.2.html). *C*, heatmap of significantly dysregulated proteins across the groups based on their relative abundance values. The 12 proteins displayed were significant in all three pairwise group comparisons, as shown in the intersection of the Venn diagram. *D*, significant canonical pathways from IPA pathway analysis of AD-DLB *versus* AD-alone. The height of the bars indicates the degree of dysregulation, with upregulated proteins shown in *red*, downregulated proteins shown in *green*, and proteins with no significant change shown in *gray*, and the remaining empty space indicates no overlap of proteins. The numbers above represent the total percentage of proteins in the pathway. The numbers towards the *right* on the end of each bar (pathway) indicate the total number of proteins involved in the pathway under homeostatic conditions. Normal (n = 6), AD alone (n = 5), and AD-DLB (n = 5).

To reveal the differences in the proteome composition of AD-DLB (fast-progressors) and AD-only/alone (slow-progressors), we isolated total protein from temporal cortices (BA20) of AD-DLB (n = 5), AD alone (n = 5), and normal cases (n = 6) and performed label-free quantitative LC-MS/MS. This label-free quantitative LC-MS/MS proteomics assessed differential protein expression across three pairwise comparisons of groups as follows: AD alone vs. Normal (AD/N), AD-DLB vs. Normal (AD-DLB/N), and AD-DLB *versus* AD alone (AD-DLB/AD alone). Differential expression was visualized using volcano plots with thresholds set at log_2_FC ≥ ±/-1.0 for fold change and -log_10_(q) ≥ 1.3 (equivalent to *p* < 0.05) for statistical significance (Fig. 4*A*). In the AD alone vs N, the upregulation of APP, GFAP, and CHL1 proteins indicates AD pathology-associated markers (Fig. 4*A* and Table S2). In the AD-DLB *versus* Normal, there is a distinct group of proteins upregulated compared to AD-alone. In the AD-DLB vs AD alone comparison, the upregulation of NIPSNAP2, MT-ND2, STOM, and ATP6V0C indicates compensatory mechanisms to deal with metabolic dysfunctions and increased demands of protein degradation in AD-DLB. In addition, the downregulation of proteins like MTCO1, GPM6A, ENPP6, and SPP1 indicates dysregulation of cellular metabolism and mitochondrial impairment (Fig. 4*A* and Table S2). Next, we assessed common proteins involved in the three pairwise comparisons of groups from our LC-MS/MS data through a Venn diagram. We found that there are 95, 96, and 51 significantly dysregulated proteins among AD/N, AD-DLB/N, and AD-DLB/AD pairwise comparisons, respectively. Among them, 12 proteins were common in all pairwise group comparisons as depicted in the intersection of the Venn diagram (Fig. 4*B*). The heatmap illustrates the differences in the expression of these 12 common, significantly dysregulated proteins among all pairwise comparisons (Fig. 4*C*).

To gain biological insights and functional relevance of differentially expressed proteins that differentiate the AD-DLB group from AD-alone and normal groups, we performed canonical ingenuity pathway analysis (IPA) on our LC-MS/MS data. Upon comparison of AD-DLB vs. AD alone through IPA, we found 48 significantly dysregulated pathways. Of these, 15 pathways are strictly related to metabolism, including respiratory electron transport, oxidative phosphorylation, mitochondrial dysfunction, pentose phosphate pathway, aspartate degradation, malate-aspartate shuttle, glutathione redox reactions, TCA cycle, and leukotriene biosynthesis (Fig. 4*D* and Fig. S5). Among all these, Aspartate degradation, Malate aspartate shuttle, and TCA cycle pathways were primarily dysregulated, showing almost 70% dysregulation. It suggests a profound impact on energy metabolism in AD-DLB disease compared to AD alone. Apart from metabolic pathways, other important pathways related to GABA synthesis and chaperone-mediated autophagy were also dysregulated in AD-DLB disease, suggesting impaired neurotransmission and increased demands to protein aggregate clearance (Fig. 4*D* and Fig. S5). We also show how these dysregulated pathways are intricately interconnected with one another (Fig. S5). The comparison between AD-DLB vs. Normal and AD alone vs. Normal from IPA analysis showed disease-specific pathway dysregulations (Figs. S6–S9). In summary, AD-DLB is distinct from its AD-alone counterpart, exhibiting unique proteomic signatures with severe metabolic and mitochondrial dysfunctions.

## Discussion

Dementia progression in AD patients is strongly associated with tau pathology but not with amyloid pathology (29, 30). However, not all patients with dementia progress at the same rate (15). It is hypothesized that this kind of heterogeneity in the progression of dementia can be due to several underlying co-pathologies (15, 17). Lewy body disease is one of the most common co-pathological associations in Alzheimer's disease (19). Our analysis of the longitudinal data revealed that nearly half of patients with AD had Lewy body pathology. Interestingly, we found that only 35% of patients with AD in the longitudinal cohort analysis had a pure or typical form of AD. After conducting survival analyses on various AD subgroups, which include AD without any co-pathology (AD-only/alone) and AD with at least one type of co-pathology, we discovered that AD-DLB and AD-TDP43 are fast-progressing dementia subgroups after the onset of mild dementia. Though AD-TDP43 progresses similarly to AD-DLB, AD-TDP43 affects less than 4% of patients with AD in our cohort analysis. Therefore, we prioritized AD-DLB cases as the focus of our further investigation. A recent study reported that soluble HMW tau and sarkosyl insoluble tau from cortical tissues of AD patients have similar tau seeding potentials (31). Our data revealed that the AD-DLB group contained more soluble oligomeric/HMW tau proteins and lower sarkosyl-insoluble tau proteins compared to AD alone, while the levels of Aβ42 were similar between the groups. However, the pathophysiological and structural characteristics of tau proteins of different molecular sizes remain unknown (Fig. 2*B*). Future studies should focus on isolating individual tau species from AD brains and characterizing their distinct properties. We further observed the presence of higher AT8 levels compared to Tau5 levels in the same cases (Fig. S3). We speculate that this may be due to the AT8 antibody-recognizing region being resistant to degradation, while the Tau5 antibody-recognizing region is degraded more quickly. However, further experiments are needed to confirm this notion.

Recent studies demonstrate that Aβ oligomers can enhance tau seeding (32). Additionally, α-Synuclein and TDP-43 interact to form hybrid fibrils (33). Our data indicate that α-Synuclein can cross-seed with tau proteins in tau FRET biosensor cells. In contrast, previous studies suggest that only tau seeds can aggregate endogenous tau in these cells (25). These differing results may be explained by our use of human brain-derived α-synuclein fibrils and commercially available α-synuclein preformed fibrils (PFFs), compared to vitro-generated α-synuclein, which may have district characteristics (25). Further, our data showed minimal co-localization and spatial overlap between Lewy body and neurofibrillary tangle (NFT) aggregates, suggesting that these two pathologies may target distinct neuronal populations with different vulnerabilities. It is possible that NFT and Lewy body aggregates may propagate in separate, non-overlapping neuronal networks. Further research should focus on the molecular profiles of neurons containing different types of intracellular aggregates. This can provide new insights into how these distinct pathologies show differential neuronal vulnerabilities and spread in AD-DLB. In addition to AD-DLB, it would be valuable to investigate the neuronal vulnerabilities and molecular profiles associated with other AD co-pathologies, such as AD-TDP43, another fast-progressing group.

Neurofibrillary tangles accumulate in excitatory neurons in Alzheimer's disease (27). However, the neuronal type in which Lewy bodies aggregate in AD-DLB remains poorly understood. Unlike in Parkinson's disease, where Lewy bodies aggregate in the cell bodies of dopaminergic neurons of the brainstem (34), Lewy bodies in AD-DLB aggregate in the neocortex, which lacks dopaminergic neuronal cell bodies. This is critical for further investigating and classifying the neuronal subtypes that aggregate Lewy bodies in the AD-DLB neocortex. Our data showed that the prefrontal cortex is more susceptible to Lewy body aggregation, while the temporal cortex is equally susceptible to NFT and LB aggregation. However, even in the brain region with equally susceptible pathologies of tau and α-synuclein, we found unique proteomic signatures with severe metabolic and mitochondrial dysfunctions present in AD-DLB compared to AD alone. In the future, it would be interesting to study the proteome of the prefrontal cortex as it is more susceptible to Lewy body aggregation in AD-DLB.

Our study provides significant contributions in the dementia progression of the AD continuum. However, we acknowledge the limitations of our study. The longitudinal study we used was specific to the AD cohort, excluding patients with DLB alone. It would be fruitful to compare all three groups, namely, AD alone, AD-DLB, and DLB alone, from the longitudinal progression of their dementia to decipher unique proteome signatures. It is also important to monitor these patients in real-time, such as through PET imaging and fluid-based biomarkers. This will help us identify new co-pathology-specific biomarkers that can help in the early diagnosis of the AD continuum.

## Conclusion

In conclusion, we report that AD-DLB is a faster progressing dementia group with more soluble tau oligomers and less insoluble tau proteins. There is selective neuronal vulnerability to neurofibrillary tangles and Lewy bodies across the neocortex. AD-DLB patients have distinct proteomic profiles that predominantly affect metabolic pathways. This study emphasizes the critical need for a deeper understanding of the pathophysiology of the AD continuum and for new biomarker discoveries that can distinguish patients with AD based on co-pathologies. Given that no treatments have been shown to prevent the onset or progression of dementia, we emphasize patient segregation based on co-pathological presentation for therapeutic studies.

## Experimental procedures

### Longitudinal data analysis with the inclusion and exclusion criteria

Longitudinal data of Alzheimer's disease patients on the MMSE (Mini-Mental State Examination) scale were obtained from the Arizona Study of Aging and Neurodegenerative Disorders/Brain and Body Donation Program (Banner Sun Health Research Institute) (35). This study adheres to the Declaration of Helsinki principles. For the analysis, patients were segregated based on the presence of any one co-pathology type in addition to AD pathology, and patients without any co-pathology were classified as having only AD (pure AD/AD-alone). In the spaghetti plot, the MMSE score at each clinic visit was plotted over time for each patient. MMSE scores of at least 25 to 20 at the patient's first clinic visit were selected as a starting point, and the rest of the patients from the cohort were not included in the analysis. A linear estimate for each group was made using simple linear regression to represent every group's average slope of MMSE decline. The groups that showed a significant deviation of the slope from zero were included in the Kaplan-Meier survival analysis of dementia. Log-rank (Mantel-Cox test) and Gehan-Breslow Wilcoxon tests were used to compare the survival curves. The age at first visit and age at death between the groups were analyzed using a Kruskal-Wallis one-way ANOVA followed by Dunn's multiple comparisons test.

### Frozen human brain post-mortem tissues

The randomly selected frozen human brain post-mortem tissues from AD-alone and AD-DLB patients (BA10) were obtained from the Banner Sun Health Research Institute. Additional *postmortem* brain tissues from BA20 and BA10 of AD, AD-DLB, and age-matched controls were received from the NIH NeuroBioBank (Human Brain and Spinal Fluid Resource Center). The cases used in this study were not pre-sorted based on race, ethnicity, gender, education, or the levels of β-amyloid and tau. The demographics of the samples used in this study are provided in Table S1.

### Gel electrophoresis and Western blot

To prepare total human brain homogenates, frozen *postmortem* brain tissues from normal, AD-alone, and AD-DLB cases were mechanically homogenized in 1× PBS (50% w/v) with a protease inhibitor cocktail (Complete Mini, Sigma Aldrich, #11836153001), and a phosphatase inhibitor cocktail (PhosSTOP EASYpack, Roche, #04906837001) using an IKA T 10 Basic Ultra-Turrax Homogenizer. Homogenization was performed at a speed setting of 3 (on a scale from 0 to 5) for 30 s to 1 min. The homogenates were then centrifuged at 15,000*g* for 20 min. The resulting supernatant was regarded as total brain homogenate (PBS-soluble), which was mixed with 2× Laemmli buffer (Bio-Rad, #1610737) in equal volumes and loaded onto a 10% SDS-PAGE gel under non-reducing conditions (without any reducing agents such as DTT or β-mercaptoethanol) to analyze differential oligomeric/high molecular weight, monomeric tau species expression and p-tau (AT8) expression. The 10% separating gel was prepared with Sodium dodecyl sulfate (Sigma Aldrich, #L3771), Acrylamide/Bis-acrylamide premix powder (ratio 29:1, Sisco Research Laboratories Pvt. Ltd, #10762), Tris HCl (Sisco Research Laboratories Pvt. Ltd, #99438), Ammonium persulfate (Sigma Aldrich, #A3678), and N,N,N′,N′-Tetraethyl ethylenediamine (Sigma Aldrich, #1.10732.0100). Following electrophoresis, proteins were transferred onto the PVDF membrane (BioRad, #1620177) and blocked with a blocking buffer containing 5% skimmed milk (HIMEDIA, #GRM1254-500G) in 1× PBST (phosphate buffer with 0.01% Tween-20 (Sigma-Aldrich, #P1379)). PVDF membranes were incubated overnight with the respective primary antibodies at 4 °C in 5% skimmed milk. The following day, membranes were washed with 1× PBST (6 times × 10 min) and incubated with respective secondary antibodies. Finally, membranes were washed again with 1× PBST (six times for 10 min each) and developed with ECL solution (BioRad, #170-5050), then imaged with Chemidoc MP imaging system (BioRad). Tau5 (Invitrogen, #AHB0042), AT8 (Invitrogen, #MN1020), secondary HRP labeled antibodies-anti-mouse HRP linked IgG (Cell Signalling Technology, #7076S) were used.

### Immunoprecipitation

AD-DLB post-mortem brain tissue (BA20) weighing 50 to 100 mg was used to immunoprecipitate α-Synuclein using the p-α-Synuclein antibody (Cell Signalling Technology, #23706S). We used a direct IP kit (Thermo Scientific, Pierce Direct IP Kit, #26148) and followed the methods as per the manufacturer's instructions. Briefly, the brain tissue was lysed in IP lysis buffer using an IKA T 10 Basic Ultra-Turrax Homogenizer. Homogenization was performed at a speed setting of 3 (on a scale from 0 to 5) for 30 s to 1 min. The resultant brain homogenate was centrifuged at 5000*g* for 10 min at 4 °C. From here, a small fraction of the resultant supernatant was saved for loading the total fraction for the Western blot to validate IP and Co-IP. The remaining resultant supernatant was subjected to direct IP protocol. IP fraction was finally eluted in 30 μl elution buffer and used for western blotting to validate IP (with α-Synuclein antibody, Invitrogen, 701085) and Co-IP (with tau5 antibody, Invitrogen, #AHB0042). The samples were treated with DTT and heated at 95 °C for 5 min for western blotting. IP fraction was also used for TEM validation without treating with any denaturing or reducing agents. For cross-seeding assay, the eluted fraction was subjected to brief sonication using a Qsonica sonicator with 20% amplitude, a total of 5 pulses (1-s pulse and 3-s rest).

### Immunocytochemistry/tau cross-seeding assay

The tau seeding assay was performed using Tau FRET biosensor cells (HEK-293), which stably express the tau repeat domain containing the P301S mutation fused with CFP and YFP (ATCC CRL-3275). Cells were cultured in 24-well plates with coverslips under standard conditions (5% CO_2_, 37 °C). To prepare 50 ml of complete medium, 44 ml of DMEM was supplemented with 5 ml of fetal bovine serum, 500 μl (100×) glutamate, 500 μl (100×) sodium pyruvate, and 500 μl(100×) Penstrep. For cross-seeding endogenous tau, immunoprecipitated α-Synuclein from AD-DLB post-mortem brain (BA20) and α-Synuclein preformed fibrils (PFFs) (ACROBiosystems, #ALN-H5115) were used. It was briefly sonicated as described previously. The α-Synuclein preparations were then mixed with 1% Lipofectamine 2000 for 30 min at room temperature before being transfected into cells at 60 to 70% confluency. Cells were incubated for 48 h under standard conditions (5% CO_2_, 37 °C). Following incubation, cells were fixed with 4% formaldehyde for 30 min. Tau aggregates were imaged using confocal microscopy with a 488 nm excitation laser at 40× magnification using Olympus FV3000. Image analysis was performed using Image J software.

### Isolation of sarkosyl-insoluble tau fractions from frozen human brain post-mortem tissues

The isolation of sarkosyl-insoluble tau was performed by following protocol (26). In brief, ∼100 mg of cortical gray matter from Brodmann area 20 was dissected using a sterile surgical blade. Nine volumes (v/w) of high-salt buffer with the composition of 10 mM Tris HCl (pH 7.4), 0.8 mM NaCl, 1 mM EDTA, 2 mM DTT, 10% sucrose, 0.1% sarkosyl, PMSF, protease inhibitor cocktail (Complete Mini, Sigma Aldrich, #11836153001), and phosphatase inhibitor cocktail (PhosSTOP EASYpack, Roche, #04906837001) were used to homogenize using an IKA T 10 Basic Ultra-Turrax Homogenizer and centrifuged at 10,000*g* for 10 min at 4 °C. After centrifugation, the pellets were re-extracted at least twice using the same high-salt buffer, and all three low-speed supernatants acquired were pooled together. An additional sarkosyl was added to this pooled extraction to make it 1%. After 1 h of nutation at room temperature, all the samples were ultracentrifuged at 300,000*g* for 1 h using a TLA 100.3 rotor in an Optima-MAX-XP ultracentrifuge from Beckman Coulter. The resulting high-speed supernatant-1 (sarkosyl-soluble fraction) was stored at −80°C for future use, and the sarkosyl-insoluble pellets-1 were washed once with cold 1× PBS, followed by resuspension in 6 volumes of 1× PBS, and ultracentrifuged at 100,000*g* for 30 min at 4 °C. Finally, pellets were mixed in 1× PBS with at least one-fifth of the pre-centrifugation volume. The resulting fractions were further purified by sonicating them with 20 to 40 pulses (1 s per pulse with 20% amplitude) using a sonicator (QSONICA). Further sonicated fractions were centrifuged again at 10,000*g* for 30 min at 4 °C to remove any large debris present. This final supernatant contained enriched PHFs and sarkosyl-insoluble tau. The purified, insoluble tau fractions were validated by immunogold TEM using the Tau5 antibody.

### Dot blot

For the dot blot, 0.5 μl (soluble and insoluble) was loaded from each sample onto the PVDF membranes, which were prewetted with methanol and left for around 1 h to allow the samples to settle. Followed by the addition of 5% skimmed milk in PBS with 0.05% Tween20 (PBST) and blocking the membranes for 30 min, then the blot was probed with primary antibodies and incubated overnight at 4 °C in 5% skimmed milk. Membranes were washed three times (10 min each) with PBST, followed by the addition of a secondary HRP-conjugated antibody for 1 h in 5% skimmed milk at room temperature. Again, membranes were washed three times (10 min each) with PBST. Finally, membranes were developed by the addition of the Clarity Western ECL substrate, Bio-Rad. Dot blots were analyzed by Image Lab software from Bio-Rad. Antibodies used were Tau5 (Invitrogen, #AHB0042), and AT8 (Invitrogen, #MN1020).

### Pronase resistance assay

A pronase resistance assay was performed using sarkosyl-insoluble brain extracts from the anterior prefrontal cortex of an AD-DLB patient (n = 2, pooled for this assay). The sarkosyl-insoluble fraction, prepared in 1× PBS, was treated with pronase enzyme (Roche, #10165921001) at a working concentration of 0.4 mg/ml. At specific time points (0, 10, 30, and 60 min), the reaction was stopped by adding an equal volume of stopping solution, composed of 2× Laemmli buffer containing 50 mM DTT and 100 mM PMSF, resulting in a final sample-to-stopping solution ratio of 1:1. The samples from each time point were then analyzed by SDS-PAGE followed by western blotting.

### A**β**42 ELISA

*Postmortem* tissues from the BA20 brain region were homogenized, and the total brain homogenate was centrifuged at 15,000*g* for 20 min at 4 °C. The resulting supernatant, considered as the total brain homogenate, was used for western blotting in this study as mentioned above. The remaining 1× PBS-insoluble pellet was processed to extract the guanidine hydrochloride (GnHCl) soluble fraction. In brief, the pellets (50% w/v) were sonicated using a Qsonica sonicator (parameters: two 10-s pulses at 50% amplitude) in 50 mM Tris-HCl buffer containing 5M guanidine hydrochloride and a protease inhibitor cocktail. Following sonication, the samples were rotated on a rotator at room temperature for 6 h to facilitate the extraction of the GnHCl-soluble fraction. The samples were then centrifuged at 15,000*g* for 20 min at 4 °C, and the supernatant was collected as the GnHCl soluble fraction isolated from the PBS-insoluble material. The total protein concentration was determined using the Bradford assay. Aβ42 levels were quantified using the human Aβ42 ELISA kit from Invitrogen (#KHB3441), following the manufacturer's instructions. The data presented in Supplementary Figure indicate the concentration of Aβ42 (pg/μg of total protein) per sample. This assay includes the cases from the BA20 brain region, with n = 7 for normal subjects and n = 10 each for AD-only/alone and AD-DLB.

### Immunohistochemistry

Blocks of gray matter from frozen human brain post-mortem tissues were cut, followed by the addition of 4% paraformaldehyde, and left for 48 h at 4 °C. Post-fixation, 30% sucrose was used as a cryoprotectant and left for 48 h at 4 °C. For sectioning, the blocks of post-mortem tissue were frozen in PolyFreeze Tissue Freezing Medium (Sigma-Aldrich, #SHH0026). Then, 30-micron slices (cryostat, Leica, #CM1520) were made for each tissue or case. Three sections, one after the other, were collected onto positively charged, PDL-coated multifrost (+) glass slides. These sections were left to air dry for 30 to 60 min, followed by blocking with 5% donkey serum (made in 0.3% Triton X 100–1X PBS) for 30 min. The sections were stained with primary antibodies in 5% donkey serum overnight at 4 °C, followed by 6 washes (each lasting 5 min) with 1× PBS. The sections were then incubated in the dark for 1 h at room temperature with secondary antibodies in 5% Donkey serum, followed by 6 washes (each lasting 5 min) with 1× PBS. The sections were then counterstained with DAPI (1:1000 in 1× PBS) for 10 min. After the final rinse with 1× PBS twice (5 min each), the sections were mounted with an antifade mounting medium (Vectashield, #H-1000). Antibodies used were AT8 (Invitrogen, #MN1020), p-α-Synuclein (Cell Signaling Technologies, #23706S), Alexa Fluor 488 (Invitrogen, #A32790), Alexa Fluor 555 (Invitrogen, #A32773).

### Confocal imaging and Imaris

Immunostained human post-mortem brain sections were imaged with 20× (air), 63× (oil), and 100× (oil) objectives under an Olympus FV3000 confocal microscope. Each case with at least three sections stained on a single multi-frost (+) glass slide was imaged randomly with at least five images per section. For images captured at 20× and 63× magnification, a step size of 2 μm was used. For images taken at 100× magnification, a step size of 1 μm was used in the Olympus FV3000 imaging software. ImageJ software was used for all the quantifications in this study. ImageJ software was also used for the co-localization analysis of p-tau/p-αsynuclein + cells. For 3D reconstruction of p-tau/p-α-synuclein co-localized cells, we used Imaris x64 10.0.1 software from Oxford Instruments.

### Immuno-electron microscopy (Ig-TEM)

The sarkosyl-insoluble fraction, resuspended in 1× PBS, was further diluted 1:1 with 1× PBS. This diluted fraction was then applied onto carbon/formvar-coated 300-mesh copper grids, which were glow-discharged before the sample addition. The sample was left on the grid for 90 to 120 s, after which any excess sample was blotted off using blotting paper. The grid was then washed twice with 1× TBS buffer (pH 7.4). Subsequently, the grid was incubated in a 1 mg/ml BSA-blocking solution for 30 min. After blocking, a primary antibody at the appropriate dilution was applied to the grid and incubated for 1 h under moist conditions. The grid was then washed three times with 1× TBS buffer. Next, a 6 nm gold-tagged secondary antibody was applied and incubated for 45 min under moist conditions, followed by six washes with 1× TBS buffer. Finally, 1% uranyl acetate was applied to the grid and immediately blotted off. The prepared grid was analyzed on a TALOS L120C Transmission Electron Microscope from Thermo Scientific, operated at 120 kV. The primary antibody used was Tau5 (Invitrogen, #AHB0042), diluted 1:40 in blocking buffer, and the secondary antibody was an anti-mouse 6 nm gold-tagged antibody (Goat Anti-Mouse IgG H&L 6 nm Gold, Abcam, #ab39614), diluted 1:20 in blocking buffer. For imaging immunoprecipitated p-α-synuclein (pS129) fraction and α-synuclein PFFs, 5 μl of IP fraction and 5 μl of 250 ng/μl PFFs fraction were directly placed on glow-discharged grid for 90 to 120 s. Any excess sample was blotted off using blotting paper. 1% uranyl acetate was applied to the grid and immediately blotted off, and the prepared grid was analyzed on a TALOS L120C Transmission Electron Microscope from Thermo Scientific, operated at 120 kV.

### Isolation of total protein from frozen human *postmortem* tissues for LC-MS analysis

Normal (n = 6), AD alone (n = 5), and AD-DLB (n = 5) post-mortem brain samples from the BA20 brain region were cut to acquire 50 mg of tissue from each case. Homogenization was performed in sample buffer (7M Urea, 2M Thiourea, 40 mM Tris, 4% CHAPS, 65 mM DTT, 1 mM EDTA, 1 mM PMSF, protease inhibitor cocktail (Complete Mini, Sigma Aldrich, #11836153001), and phosphatase inhibitor cocktail (PhosSTOP EASYpack, Roche, #04906837001) with 50% w/v, followed by sonication using a sonicator (QSONICA). Samples were then centrifuged at 15,000*g* for 20 min. The supernatant was collected and subjected to buffer exchange (50 mM ammonium bicarbonate) using a centrifugal filter with a molecular weight cutoff (MWCO) membrane of 3 kD (Merck-Millipore). A Bradford assay was performed, and the samples' concentrations were interpolated using a standard curve. The samples were adjusted to a protein concentration of 1 μg/μl in 50 mM ammonium bicarbonate buffer and subsequently sent for LC-MS/MS separation and analysis to the DBT-SAHAJ National Facility for Mass Spectrometry-based Proteomics, RGCB, Trivandrum.

### LC-MS/MS data analysis and pathway enrichment

We analyzed the proteins enlisted from the LC-MS/MS data of AD-DLB, AD-alone, and normal samples by categorizing them based on their abundance ratio in three different groups: AD-DLB/AD, AD-DLB/Normal, and AD/Normal. For each pairwise group comparison, the significance threshold was set for their abundance adjusted *p*-value (with Benjamini-Hochberg correction) being less than 0.05 for all these analyses. We manually calculated abundance ratios using the raw abundance values provided for each protein. The volcano plots were generated by VolcaNoseR package (https://huygens.science.uva.nl/VolcaNoseR/). For volcano plots, thresholds were set at log_2_FC ≥ ±1.0 for biologically significant changes and -log_10_(q) ≥ 1.3 (q < 0.05) for statistical significance. Additionally, we looked for proteins commonly expressed in all the pairwise comparisons of groups mentioned above using Venny 2.0 (27120465). The heatmap was generated using GraphPad Prism 9. All 12 proteins shown in the heatmap are statistically significant below adjusted *p* value < 0.05 across all pairwise comparisons of groups. These proteins are shown in the intersection of all pairwise comparisons of groups in the Venn diagram. ^33^To explore the possible significant pathways of the differentially expressed proteins, we used the Ingenuity Pathway Analysis tool (Qiagen) by using manually calculated abundance ratios for each pairwise comparison (*e.g.*, AD-DLB/AD alone). This analysis yielded several canonical pathways of these differentially expressed proteins across all pairwise comparisons of groups. We used more stringent biological filters towards Homo Sapiens and less stringent (flexible filters) for the central nervous system and its cell lines for all pairwise comparisons of groups (*e.g.*, AD-DLB/AD alone). The canonical pathways with -log (adjusted *p*-value) ≥ 1.3 were considered for their significance. Connectivity among these enlisted pathways was also found across all pairwise comparison of groups.

### Data analysis and statistics

All information regarding *p*-values and the type of statistical test used is mentioned in the respective figure legends. A *p*-value < 0.05 was considered significant. All statistical analyses were performed using GraphPad Prism software. To check the normality of the data, we used the Shapiro-Wilk test (a *p*-value below 0.05 was considered a deviation from normality). An unpaired Student's *t* test with and without Welch's correction and a non-parametric *t* test (Mann-Whitney test) were used to compare two groups. One-way analysis of variance (ANOVA) with Welch's correction and non-parametric Kruskal-Wallis ANOVA were used to compare more than two groups. For survival analysis, the Kaplan-Meier survival curve with a 10-point MMSE drop from mild dementia (MMSE: 20–25) was used. For comparison of the survival curves, the log-rank (Mantel-Cox) test and the Gehan-Breslow-Wilcoxon test were used for the statistics of the survival curve. For Western blot quantification, ImageJ software was used to select the interested immunoreactive band with a rectangular tool, and a wand tool was used to quantify the area under the curve of the immunoreactive band. For normalization with total protein, a PVDF membrane stained with Amido black (Amido black staining solution, Sigma Aldrich, #A8181) was used for the quantification.

## Data availability

The manuscript contains all of the data analyzed in this study.

## Consent for publication

All authors have approved the manuscript and agree with its submission.

## Supporting information

This article contains supporting information.

## Conflict of interest

The authors declare that they have no conflicts of interest with the contents of this article.

## Supporting information

Supplementary table 2

Suppl info R2
