# Supplementary material for: Distinct neuronal vulnerability and metabolic dysfunctions are characteristic features of fast-progressing Alzheimer's patients with Lewy bodies
Source: J Biol Chem. 2025 Mar 10;301(4):108396. doi: 10.1016/j.jbc.2025.108396 (PMC12002820; doi:10.1016/j.jbc.2025.108396)
Supplement: Suppl info R2 [file mmc3.docx]

**SUPPLEMENTARY FIGURE-1**


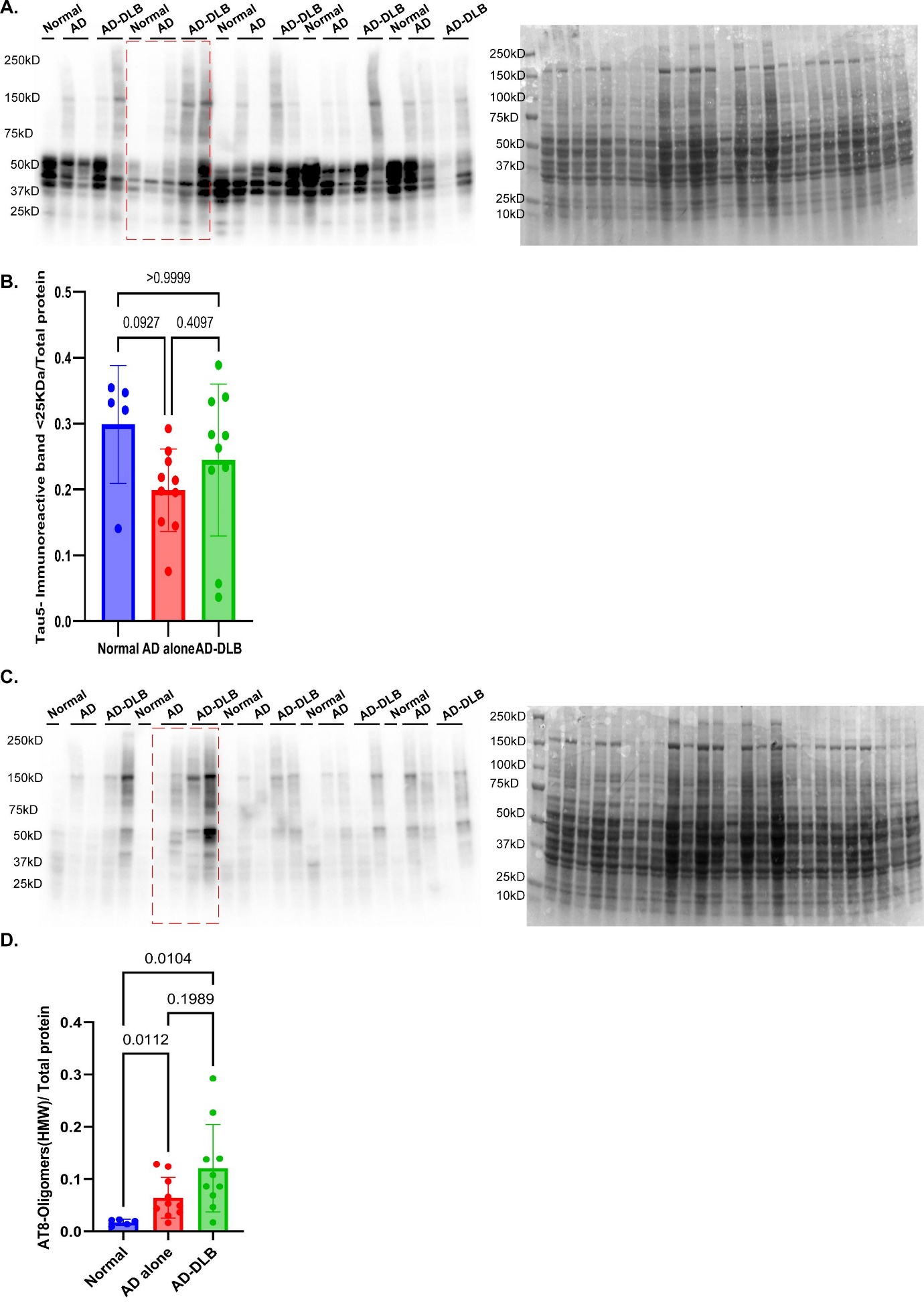


**SUPPLEMENTARY FIGURE-2**


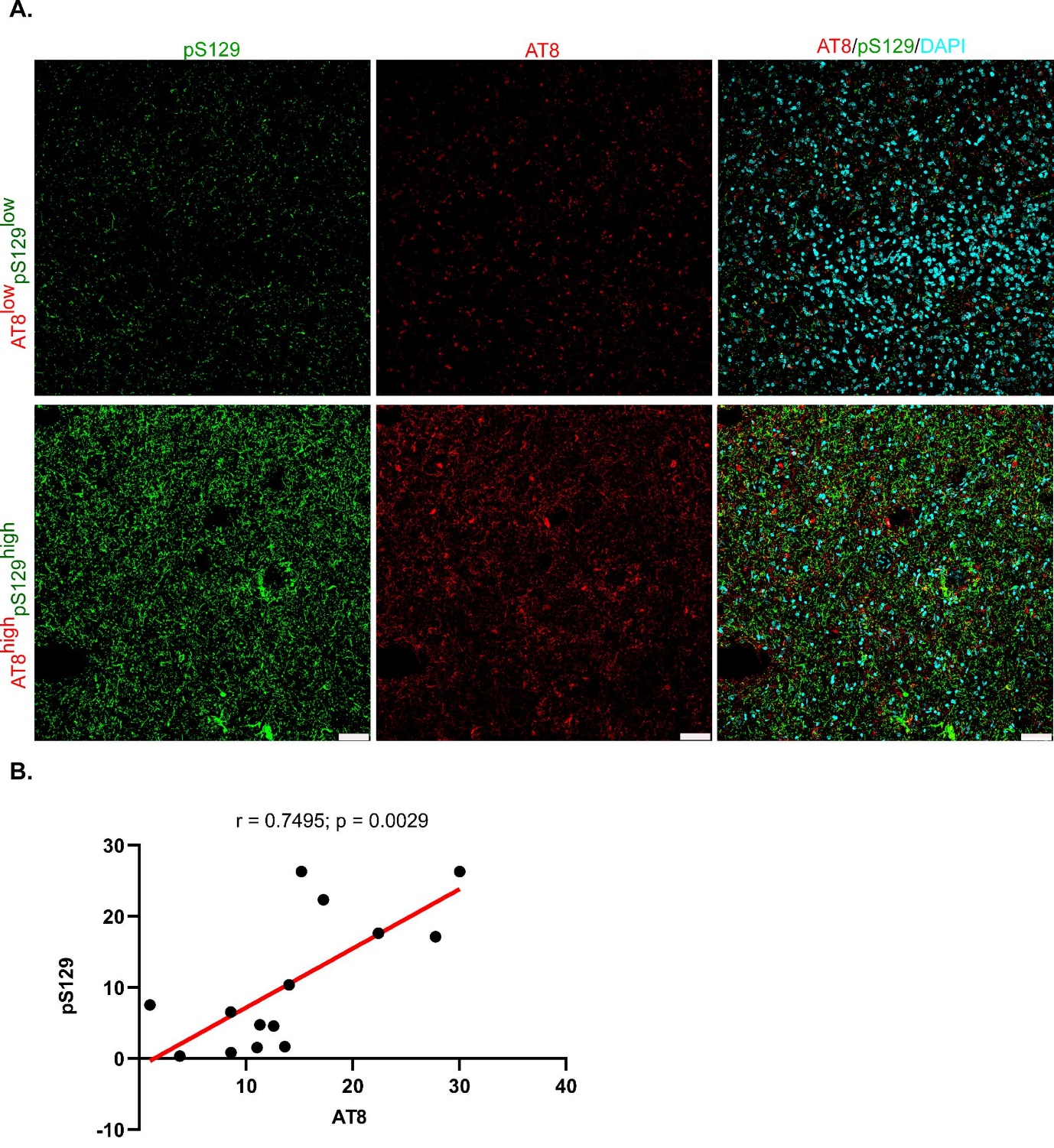


**SUPPLEMENTARY FIGURE-3**


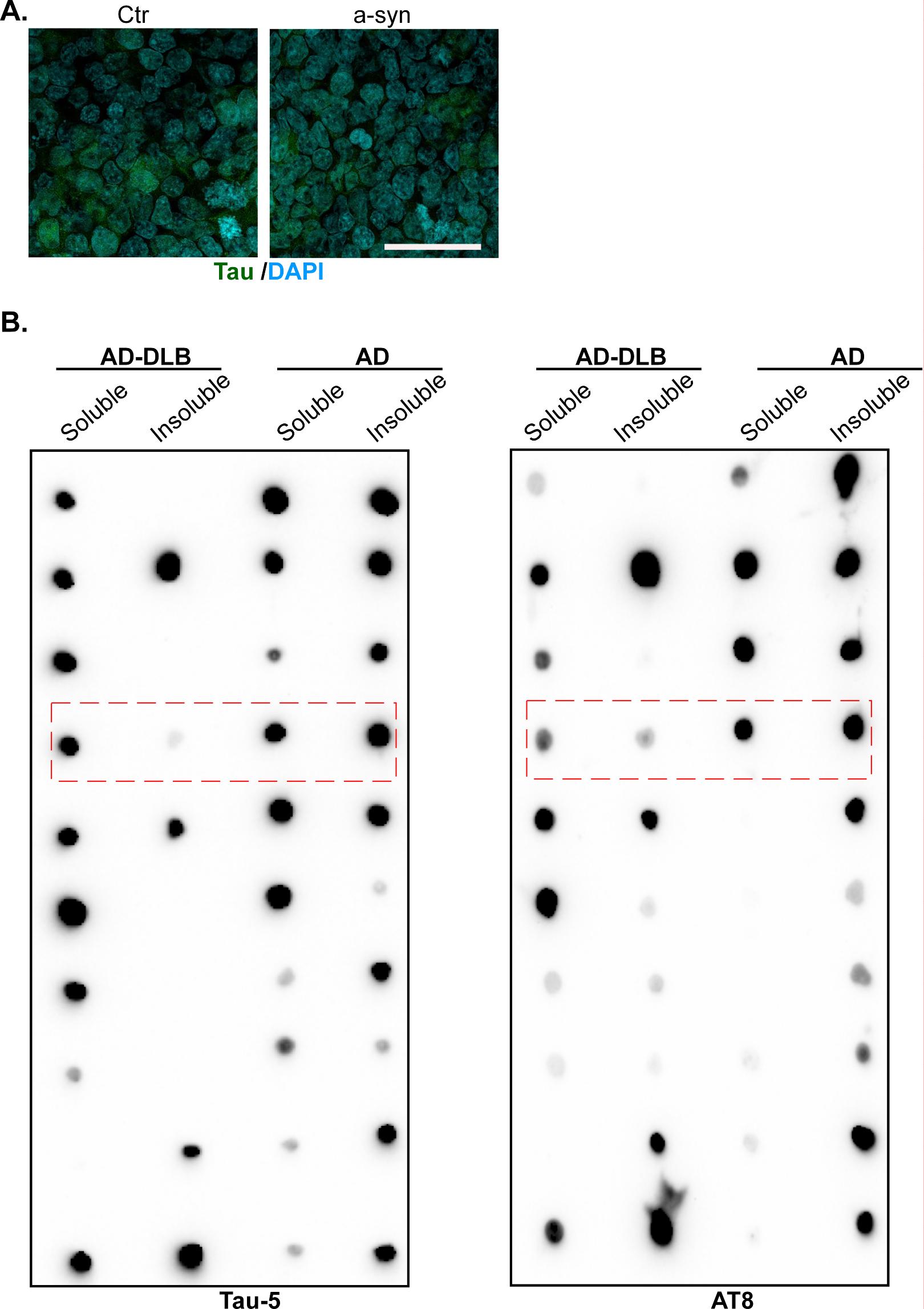


**SUPPLEMENTARY FIGURE-4**

**
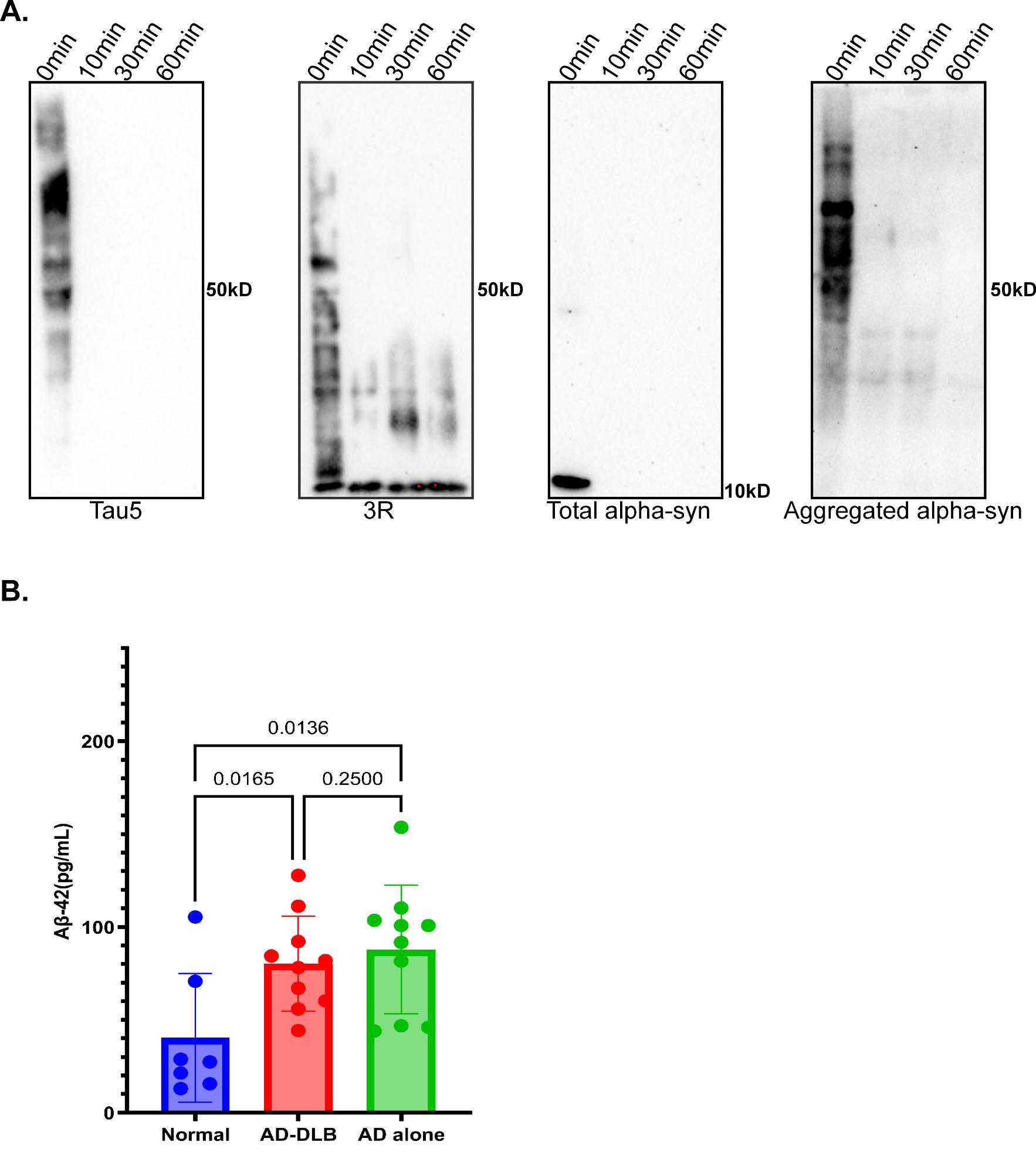
**

**SUPPLEMENTARY FIGURE-5**

**
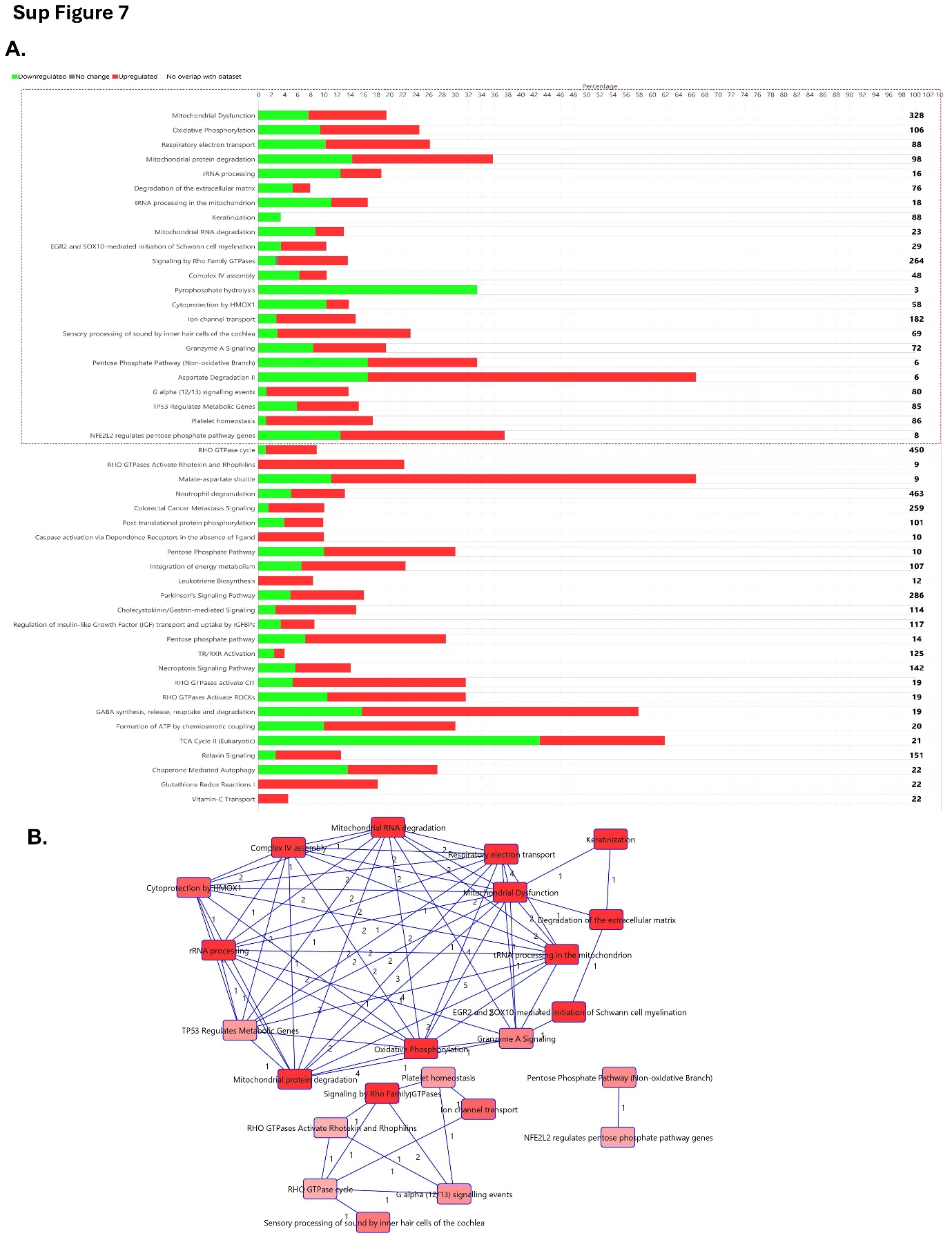
**

**SUPPLEMENTARY FIGURE-6**

**
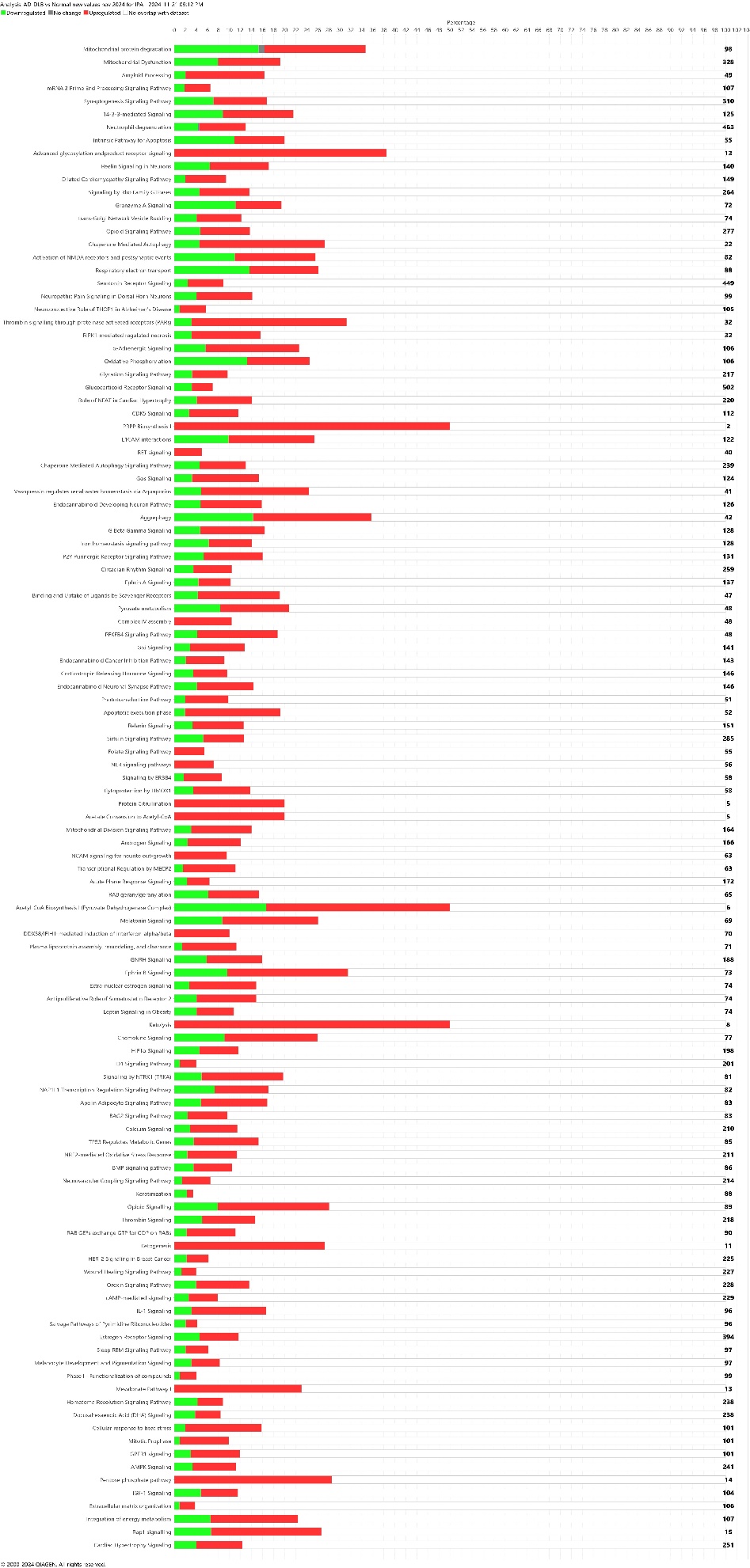
**

**SUPPLEMENTARY FIGURE-7**

**
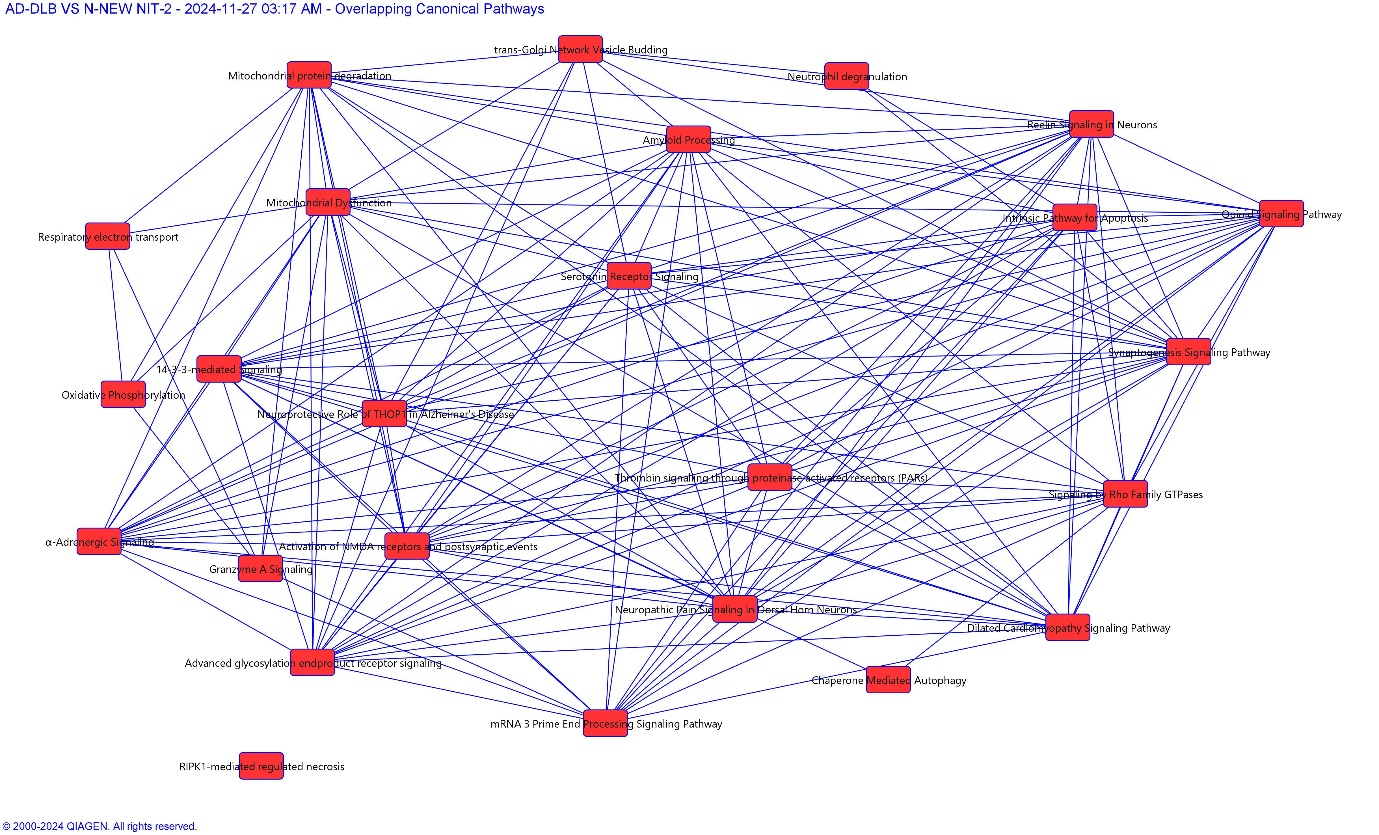
**

**SUPPLEMENTARY FIGURE-8**

**
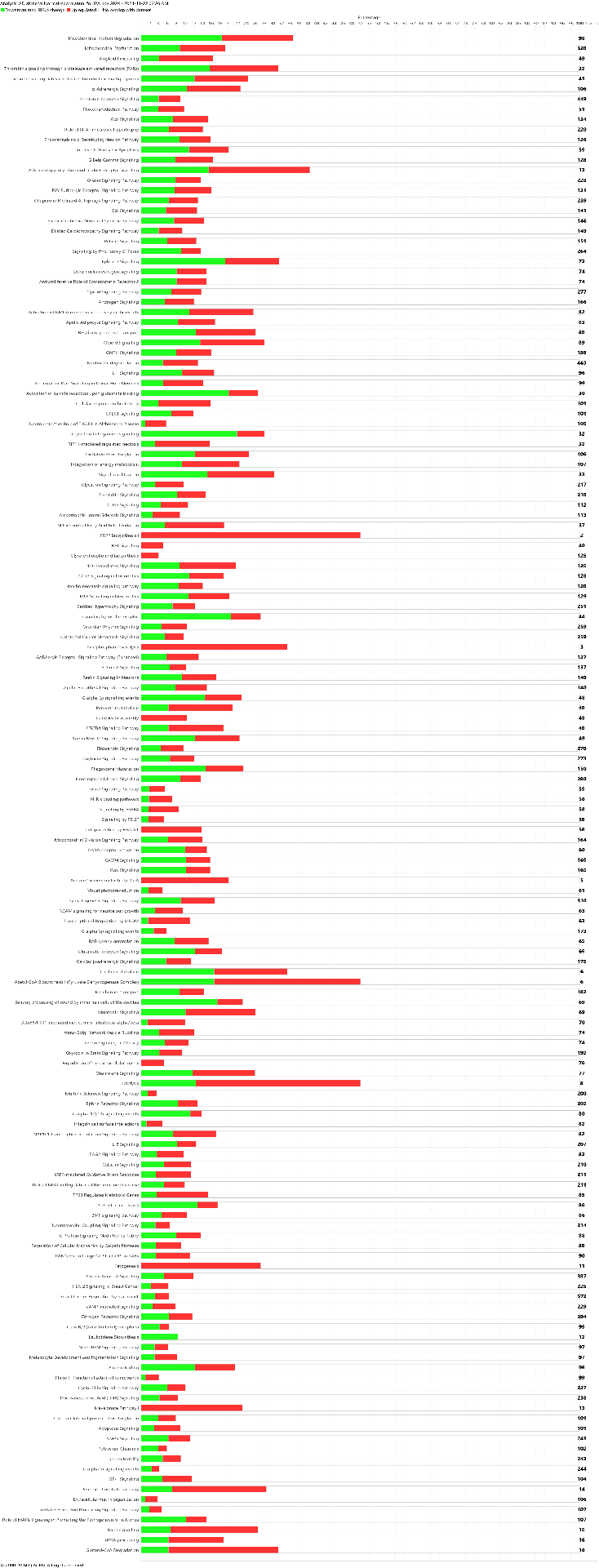
**

**SUPPLEMENTARY FIGURE-9**

**
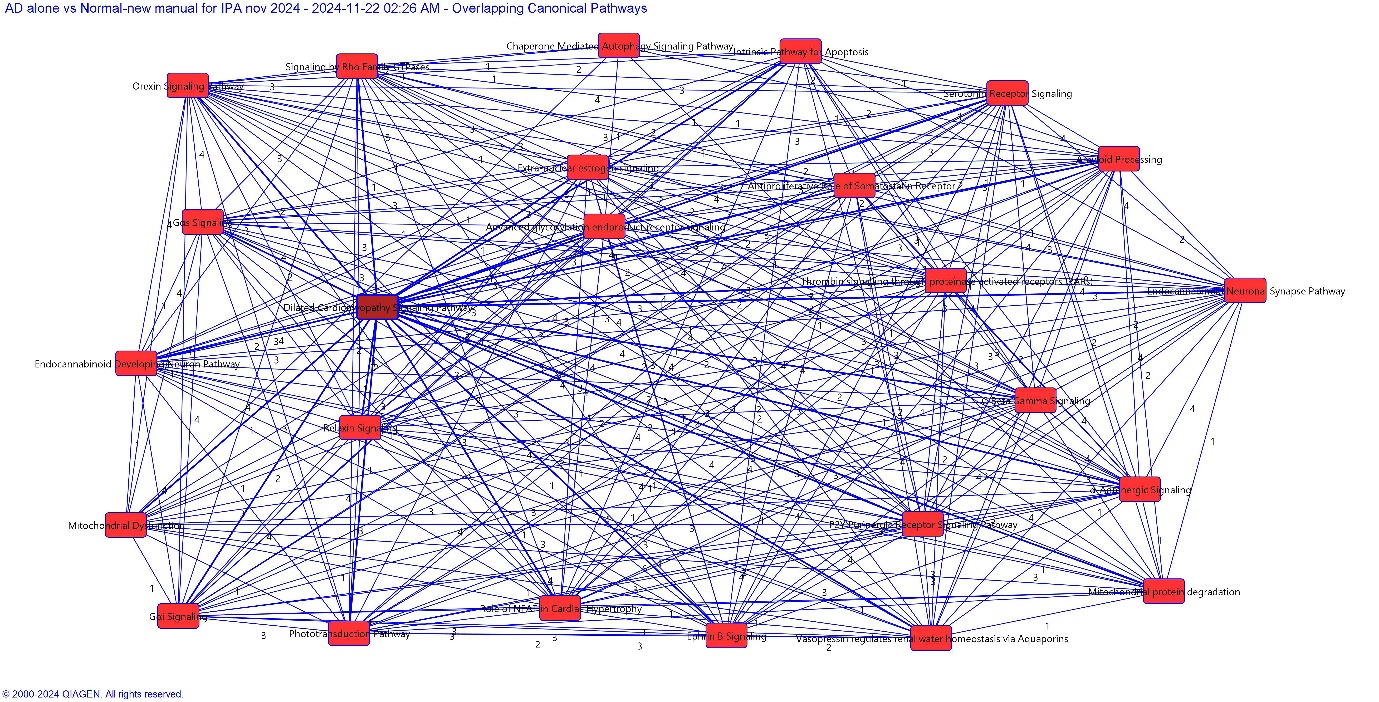
**

**Supplementary Figure-1: A.** SDS-PAGE was performed using PBS-soluble fractions. The blot was subsequently probed with a Tau-5 antibody, and the same membrane was stained with an amido black solution for total protein quantification. **B**. To compare three groups (Normal, AD alone, AD-DLB) for Tau5- immunoreactive band 25kD and below, statistical analysis was conducted using Kruskal-Wallis test (p = 0.0780), followed by Dunn’s multiple comparisons test, comparing the mean of each group with every other group. **C.** SDS-PAGE was performed using PBS-soluble fractions. The blot was probed with an AT8 antibody, and the same membrane was stained with 1X amido black solution for total protein quantification. **D.** To compare three groups (Normal, AD alone, AD-DLB), statistical analysis was conducted on the AT8 blot using the Brown-Forsythe ANOVA test (p = 0.0069) and Welch’s ANOVA test (p = 0.0007), followed by Dunnett’s T3 multiple comparison test, comparing the mean of each group with every other group. Normal (n=5), AD alone (n=10), and AD-DLB (n=10).

**Supplementary figure-2: A.** Immunostaining with AT8 and pS129 antibodies and subsequent Pearson correlation analysis (**B**) revealed that the levels of α-synuclein pathology are positively associated with the levels of tau pathology in the BA20 brain region. In Fig B, the top panel represents an AD-DLB case where AT8 and p-α-synuclein expressions are low, while the bottom panel represents an AD-DLB case where both AT8 and p-α-synuclein expressions are high. scale bar = 50 µm. AD-DLB cases, n= 14; BA20 brain region.

**Supplementary figure-3: A.** α-synuclein, which was prepared as described in reference 25, did not induce tau aggregates in tau FRET biosensor cells. scale bar = 50 µm **. B.** Dot blots were performed using sarkosyl-soluble and insoluble fractions from AD alone and AD-DLB and probed with Tau-5 and AT8 antibodies, respectively. AD alone (n=9) and AD-DLB (n=9). The case number 9 was excluded since there were no signals.

**Supplementary figure-4: A.** Sarkosyl-insoluble fractions from AD-DLB were treated with pronase enzyme (0.4 mg/ml) for varying time points. Within 10 minutes, the bands in the Tau5 blot were no longer visible, and total α-synuclein was fully degraded. Aggregated α-synuclein showed resistance to pronase, as they were only faintly detectable in the aggregated α-synuclein blot at both the 10- and 30-minute time points. However, these aggregates were more susceptible to degradation than the tau core regions (3R) as seen in the 3R blot. This suggests that in the AD-DLB brain, the tau fibril core region (3R) is more resistant to degradation. **B.** The Aβ42 ELISA was performed using the Human Aβ42 ELISA kit from Invitrogen (#KHB3441). Soluble fractions of guanidine hydrochloride (GnHCl) buffer from PBS-insoluble fractions of all cases (BA20 brain region) were prepared. Total protein concentration was estimated using the Bradford assay, followed by Aβ42 quantification using ELISA. The y-axis represents the picograms (pg) of Aβ42 per microgram of total protein from the GnHCl soluble fraction. The Kruskal-Walli’s test showed a significant difference in Aβ42 levels between the groups (p = 0.0318). Pairwise comparisons showed significant differences in Aβ42 levels between Normal and AD-only/alone (adjusted p-value (q-value) = 0.0136), as well as between Normal and AD-DLB (adjusted p-value (q-value) = 0.0165). However, no significant difference was found between AD-DLB and AD-only/alone (adjusted p-value (q-value) = 0.2500). These analyses were conducted using the Two-Stage Linear Step-Up Procedure of Benjamini, Krieger, and Yekutieli to correct for multiple comparisons and control the false discovery rate (FDR). Normal (n=7), AD-only/alone (n=10), and AD-DLB (n=10).

**Supplementary figure-5: A.** Canonical pathways from Ingenuity Pathway Analysis (IPA) of the AD-DLB vs. AD alone comparison (AD-DLB/AD alone) based on LC-MS/MS data. Only significant pathways with a -log10(adjusted p-value/q-value) ≥ 1.3 (equivalent to p = 0.05) are shown. Numbers above represent the total percentage of proteins in the pathway. The height of the bars indicates the degree of dysregulation, with upregulated proteins shown in red, downregulated proteins shown in green, and proteins with no significant change shown in grey, and the remaining empty space indicate no overlap of proteins in that pathway from our proteomics data. The numbers towards the right on the end of each bar (pathway) indicate the total number of proteins involved in the pathway under homeostatic conditions. The highlighted boxes in this supplementary figure are shown as representative images in Figure 4D. The red boxed area is shown Figure 4. **B.** Interaction network illustrating the relationships between all significant canonical pathways from the IPA pathway analysis in the AD-DLB/AD alone comparison. The numbers on the connecting lines indicate the total number of common proteins involved in the interactions between the pathways. The sample size for LC-MS/MS: normal (n = 6), AD alone (n = 5), and AD-DLB (n = 5) from the temporal cortex (BA20).

**Supplementary figure-6:** Canonical pathways from Ingenuity Pathway Analysis (IPA) of the AD-DLB vs. Normal comparison (AD-DLB/Normal) based on LC-MS/MS data. Only significant pathways with a -log10(adjusted p-value/q-value) ≥ 1.3 (equivalent to p = 0.05) are shown. Numbers above represent the total percentage of proteins in the pathway. The height of the bars indicates the degree of dysregulation, with upregulated proteins shown in red, downregulated proteins shown in green, and proteins with no significant change shown in grey, and the remaining empty space indicate no overlap of proteins in that pathway from our proteomics data. The numbers towards right on the end of each bar (pathway) indicate the total number of proteins involved in the pathway under homeostatic conditions.

**Supplementary figure-7:** Interaction network illustrating the relationships between all significant canonical pathways from the IPA pathway analysis in AD-DLB/Normal comparison. The numbers on the connecting lines indicate the total number of common proteins involved in the interactions between the pathways. The sample size for LC-MS/MS: normal (n = 6), AD alone (n = 5), and AD-DLB (n = 5) from the temporal cortex (BA20).

**Supplementary figure-8:** Canonical pathways from Ingenuity Pathway Analysis (IPA) of the AD alone vs. Normal comparison (AD alone/Normal) based on LC-MS/MS data. Only significant pathways with a -log10(adjusted p-value/q-value) ≥ 1.3 (equivalent to p = 0.05) are shown. Numbers above represent the total percentage of proteins in the pathway. The height of the bars indicates the degree of dysregulation, with upregulated proteins shown in red, downregulated proteins shown in green, and proteins with no significant change shown in grey, and the remaining empty space indicating no overlap of proteins in that pathway from our proteomics data. The numbers towards right on the end of each bar (pathway) indicate the total number of proteins involved in the pathway under homeostatic conditions.

**Supplementary figure-9:** Interaction network illustrating the relationships between all significant canonical pathways from the IPA pathway analysis in AD alone/Normal comparison. The numbers on the connecting lines indicate the total number of common proteins involved in the interactions between the pathways. The sample size for LC-MS/MS: normal (n = 6), AD alone (n = 5), and AD-DLB (n = 5) from the temporal cortex (BA20).

**Supplementary video:** Imaris: A reconstructed 3D image illustrating the distinct spatial distribution of AT8 (red) and pS129 (green) in a co-localised cell from AD-DLB.

**Supplementary Table-1:** Subject demographics of the post-mortem tissues used in the study. This includes their gender, diagnosis, post-mortem interval (PMI), and the brain structure received.

**Supplementary Table-2:** It includes 3 sheets. (1) LC-MS/MS result interpretation, (2) LC-MS/MS results and calculations, (3) Top upregulated and downregulated proteins are enlisted from all pairwise comparisons of the groups. This includes the Log2Fold change (Log2FC) along with the protein name.
